# Comparative analysis of binding patterns of MADS-domain proteins in *Arabidopsis thaliana*

**DOI:** 10.1186/s12870-018-1348-8

**Published:** 2018-06-25

**Authors:** Niels Aerts, Suzanne de Bruijn, Hilda van Mourik, Gerco C. Angenent, Aalt D. J. van Dijk

**Affiliations:** 1 Bioscience, Wageningen UR, Droevendaalsesteeg 1, Wageningen, The Netherlands; 2 Plant-Microbe Interactions, Utrecht University, Padualaan 8, Utrecht, The Netherlands; 3 Laboratory of Molecular Biology, Wageningen University, Wageningen, The Netherlands; 4 Biometris, Wageningen UR, Droevendaalsesteeg 1, Wageningen, The Netherlands; 5 Bioinformatics, Wageningen UR, Droevendaalsesteeg 1, Wageningen, The Netherlands

**Keywords:** MADS-domain proteins, CArG-box, ChIP-seq, Transcription factor binding specificity, Sequence conservation

## Abstract

**Background:**

Correct flower formation requires highly specific temporal and spatial regulation of gene expression. In *Arabidopsis thaliana* the majority of the master regulators that determine flower organ identity belong to the MADS-domain transcription factor family. The canonical DNA binding motif for this transcription factor family is the CArG-box, which has the consensus CC(A/T)_6_GG. However, so far, a comprehensive analysis of MADS-domain binding patterns has not yet been performed.

**Results:**

Eight publicly available ChIP-seq datasets of MADS-domain proteins that regulate the floral transition and flower formation were analyzed. Surprisingly, the preferred DNA binding motif of each protein was a CArG-box with an NAA extension. Furthermore, motifs of other transcription factors were found in the vicinity of binding sites of MADS-domain transcription factors, suggesting that interaction of MADS-domain proteins with other transcription factors is important for target gene regulation. Finally, conservation of CArG-boxes between *Arabidopsis* ecotypes was assessed to obtain information about their evolutionary importance. CArG-boxes that fully matched the consensus were more conserved than other CArG-boxes, suggesting that the perfect CArG-box is evolutionary more important than other CArG-box variants.

**Conclusion:**

Our analysis provides detailed insight into MADS-domain protein binding patterns. The results underline the importance of an extended version of the CArG-box and provide a first view on evolutionary conservation of MADS-domain protein binding sites in *Arabidopsis* ecotypes.

**Electronic supplementary material:**

The online version of this article (10.1186/s12870-018-1348-8) contains supplementary material, which is available to authorized users.

## Background

Correct spatial and temporal programming of flowering and flower development is of vital importance for plant reproduction. A major part of this programming is regulated by a class of transcription factors called MADS-domain transcription factors. Members of this transcription factor family play key roles in different aspects of development and have homologs in numerous other organisms in the plant, fungus and animal kingdom [1].

In *Arabidopsis thaliana*, MADS-domain proteins can be divided into two major clades based on their conserved domains. Type I MADS-domain proteins only have their DNA-binding MADS domain in common. In contrast, the much better characterized type II MADS-domain proteins have four domains in common; the MADS domain is involved in DNA binding, the intervening domain has a role in dimerization, the keratin-like domain, also known as K-box, has a role in dimerization as well as in other protein-protein interactions, and the C-terminal domain has diverse functions, such as stabilizing protein complexes and activating transcription [2].

Over the past decades the role of MADS-domain proteins in *Arabidopsis thaliana* flower development has been extensively studied. This has led to the development of the so-called ABC(D)E model. According to this model a flower can be seen as a collection of four whorls. From the outside to the inside these whorls consist of sepals, petals, stamens and carpel(s) respectively. The identity of these whorls is determined by expression of specific genes, which can be divided into four different classes (A, B, C and E). Sepal identity is determined by expression of the *Arabidopsis* A class genes *APETALA1* (*AP1*) and *APETALA2* (*AP2*). Petal development requires combined expression of these A class genes and of B class genes, consisting of *APETALA3* (*AP3*) and *PISTILLATA* (*PI*). Stamen development requires combined expression of these B class genes and the C class gene *AGAMOUS* (*AG*). Finally, carpel identity is determined by the expression of the C class gene *AG* alone. The activity of the D class genes specifies ovule identity. Additionally, expression of genes of the E class, which consists of *SEPALLATA1,2,3* and *4* (*SEP1–4*), is required in every whorl for proper flower development. With the exception of *AP2*, the genes of all floral organ classes encode MADS-domain proteins.

Plant MADS-domain proteins bind DNA as homo- or heterodimers [3, 4]. Additionally, they can form higher-order complexes that can bind DNA at multiple sites, resulting in a DNA loop between the sites [5–7]. It is believed that the MADS-domain proteins that determine flower identity form tetrameric complexes, also referred to as ‘floral quartets’, which consist of the appropriate A, B and/or C class proteins as well as a SEP protein. This SEP protein can be seen as a molecular glue holding the tetrameric complex together [8].

Apart from regulating the identity of floral organs, MADS-domain transcription factors also have a role in the regulation of flowering, the transition from vegetative to reproductive growth. For example, SUPPRESSOR OF OVEREXPRESSION OF CONSTANS1 (SOC1) has been identified as a major hub in a gene regulatory network that regulates the timing of flowering [9]. Other examples of flowering regulators are the MADS-domain proteins FLOWERING LOCUS C (FLC) and SHORT VEGETATIVE PHASE (SVP), which repress flowering by binding as a complex of the two proteins or separately to distinct promoter sequences [10].

Each MADS-domain TF, either as a dimer or higher order complex, should regulate specific sets of target genes to control the different developmental processes in which they are involved. This requires specificity and affinity of these proteins to certain DNA sequences. MADS-domain proteins are known to bind to a DNA motif called the CArG-box, which has the consensus sequence CC(A/T)_6_GG [11]. Apart from this consensus, which will henceforth be referred to as the perfect CArG-box, the variants CC(A/T)_7_G and C(A/T)_8_G are also recognized as important for binding of some MADS-domain proteins [11, 12].

A popular technique to study binding of transcription factors to specific DNA sequences in vivo is chromatin immunoprecipitation followed by deep sequencing (ChIP-seq). ChIP-seq has been used to study DNA binding of different proteins related to flower development, among which are AG [13], AP1 [14, 15], AP3 [16], FLC [10], PI [16], SEP3 [15, 17], SOC1 [9] and SVP [10]. In each of these studies, analysis of the DNA sequences to which the proteins bound using de novo motif discovery revealed a motif that was similar to the canonical CArG-box. When comparing the CArG-box like motifs found in the different studies, differences between the motifs bound by different proteins can be observed. For example, Wuest et al. found that the AP3/PI dimer binds mainly to a motif that is reminiscent of the canonical CArG-box with three adenines at the 3′ side [16], whereas Pajoro et al. found that SEP3 binds to a similar motif, with two additional thymines at positions − 3 and − 2 before the first cytosine of the motif [15]. Based on these observations it is tempting to speculate that the observed variation in binding motifs explains the binding specificities of the different MADS-domain transcription factors. However, the methods used to analyze the ChIP-seq datasets were not the same in these studies, making it impossible to draw reliable conclusions.

In the present study, the ChIP-seq datasets of AG [13], AP1 [15], AP3 [16], FLC [10], PI [16], SOC1 [9], SVP [10] and SEP3 [15] were re-analyzed in a uniform manner. The analysis revealed that the binding motifs of the proteins were highly similar, but not identical. Furthermore, the relative importance of different features of the motif for protein binding was determined, showing that a perfect CArG-box with two or three additional adenines is likely to be the most important motif for all proteins. Additionally, non-CArG-box motifs were found with de novo motif discovery, suggesting that some MADS-domain proteins regulate their targets indirectly through interactions with other classes of transcription factors. Finally, the evolutionary importance of CArG-boxes was assessed by analyzing their conservation in different *Arabidopsis thaliana* ecotypes.

## Methods

### ChIP-seq data processing

Raw reads were downloaded from the NCBI Gene Expression Omnibus database (https://www.ncbi.nlm.nih.gov/geo/). Accession numbers were GSE45938 (AGAMOUS), GSE46986 (APETALA1 and SEPALLATA3), GSE38358 (APETALA3 and PISTILLATA), GSE54881 (FLOWERING LOCUS C and SHORT VEGETATIVE PHASE) and GSE45846 (SUPPRESSOR OF OVEREXPRESSION OF CONSTANS1). For AP1 and SEP3 the dataset from 4 days after induction was chosen for analysis. Reads were mapped to the unmasked *Arabidopsis thaliana* TAIR10 genome (TAIR10_chr_all.fas; ftp.arabidopsis.org) using SOAPaligner release 2.21 (http://soap.genomics.org.cn/soapaligner.html) with default settings, except the setting -r 0. This setting was chosen so that repeat hits, which cannot be reliably assigned to a specific part of the genome, were ignored for the analysis.

Peak calling was done for each biological replicate (if available) using the R package CSAR [18, 19]. If a biological replicate consisted of several technical replicates, the technical replicates were merged. Default settings were used, except in the mappedReads2Nhits function, in which uniquePosition was set to TRUE. This setting was chosen to ensure that only reads that did not completely overlap with other reads (suggesting they were actually the same reads that were amplified by PCR) were used for the analysis. In the ChIPseqScore function the Poisson distribution was used for scoring of enrichment. In the sigWin function a threshold of *t* = 1.3 (corresponding to a *p*-value of roughly 0.05) was used and an FDR threshold of 0.001 was taken to select significant peaks. The biological replicate with the most peaks was used for further analysis.

For further analysis, the peak center (defined as the 500 bp centered around the peak summit) was used. A custom python script was written to see if these peak centers showed any overlap between datasets.

### De novo motif discovery

De novo motif discovery was carried out using MEME-ChIP [20]. This program consists of several sub-programs that each perform a specific analysis. In the present study MEME, FIMO and CentriMO were used. MEME [21] looks for overrepresented motifs in a set of sequences compared to a background model of nucleotide frequencies in those sequences. In the present study, 500 bp sequences corresponding to the peak centers of each dataset were given as input. The central 100 bp are used by the program to look for overrepresented motifs and determine nucleotide frequencies. After finding motifs, FIMO [22] is used to find all occurrences of each motif in the 500 bp sequences provided. Finally, CentriMO [23] is used to test if the motifs found are centrally enriched. In the present study, motifs were investigated if they resembled a CArG-box or if they were in the top three motifs as defined by MEME-ChIP. Motif sequence logos were visualized using WebLogo 2.8.2 [24].

In sequences used as input for MEME-ChIP, interspersed repeats and low-complexity DNA were masked using RepeatMasker (www.repeatmasker.org) with the abblast engine, default speed and *Arabidopsis thaliana* as the DNA source. Default settings for MEME were used, with the following exceptions: Since CArG-boxes are often located close to each other, the setting -meme-mod anr was used, which assumes zero, one or multiple motif occurrences per sequence. Also, −meme-nmotifs, the amount of motifs MEME tries to find, was set to 10 to ensure that all possible significant motifs would be found. -meme-maxsites, the maximum amount of occurrence of a motif that MEME considers, was set to 5000 to ensure that all occurrences of a given motif would be found. -nmeme, the amount of sequences that MEME analyses, was set to 10,000,000 and -meme-maxsize, the total dataset size that MEME analyses, was set to 1,000,000,000,000 so that the entire dataset would be analyzed.

In an alternative approach, a custom Python script was written that analyzed the occurrence of each possible string of 10 characters long, in which each position is either defined as one of the four nucleotides (“A”, “C”, “G” or “T”) or any of the four nucleotides (“N”). Matches to these strings were searched in the same DNA sequences that were used for MEME-ChIP. The average distance between matches to these regular expressions and the closest peak summit was analyzed, and the regular expressions were ranked from lowest to highest average distance. Only regular expressions with an average distance to the peak summit of 90 bp or less and with 80 or more matches were selected for further analysis. A manual analysis was performed on this dataset to find motifs that were different from the CArG-box or secondary motifs found with MEME-ChIP analysis.

### Motif frequency and central enrichment analysis

To compare relative frequencies, the frequency of stretches of peak centers that contained a certain motif was calculated and divided by a background frequency. Two different definitions of background were used: (i) the background frequency was calculated from 100,000 randomly selected stretches of 500 bp from the entire *Arabidopsis* TAIR10 genome; or (ii) the background frequency was calculated from 100,000 randomly selected stretches of 500 bp from *Arabidopsis* promoter regions. These regions were made by concatenating the 500 bp upstream of each transcription start site, as these regions are the richest in functional motifs [25] and a large proportion of the peak centers analyzed overlap with these regions (Additional file 1: Table S1). In addition, it was calculated how many peaks in each dataset contained the motif CC(A/T)_6_GG, CC(A/T)_7_G, C(A/T)_8_G once or multiple times, and how many peaks contained a combination of two of these three motifs. For this last number, an expected value was also calculated by multiplying the frequency of peaks that contained each motif and multiplying that number with the total amount of peaks in each dataset.

To analyze central enrichment, the average distance between each match to a motif in a peak and the peak summit of that peak was calculated. For visualization of central enrichment, a kernel density estimation of the dataset containing al matches to the motif was obtained using the R function ‘density’.

### Extension analysis of CArG-box like motifs

Two different custom Python scripts were written to analyze the nucleotides around CArG-boxes. The first script looks at the frequency of different combinations of three nucleotides at both sides of a CArG-box as defined by MEME-ChIP. First, given the strand provided by MEME-ChIP, position 1 and 10 of a CArG-box was defined by comparing a CArG-box like sequence to the canonical CArG-box definition (CC(A/T)_6_GG). Position 1 was defined as the nucleotide that corresponds to the first C of the canonical CArG-box and position 10 was defined as the nucleotide that corresponds to the last G of the canonical CArG-box. The occurrence of different combination of three nucleotides at the 5′ side of position 1 (from hereon 5′ extensions) and the 3′ side of position 10 (from hereon 3′ extensions) and combinations of 5′ and 3′ extensions were counted. For the combinations, an expected value was also calculated by multiplying the occurrence of the 5′ and 3′ extensions and dividing it by the total amount of CArG-boxes. Note that 5′ and 3′ are defined here with respect to the CArG-box orientation as defined by MEME-ChIP, which is oriented to align as much adenines as possible.

A second custom Python script was written to analyze specifically extensions around the sequence NCC(A/T)_6_GGN (hence using a more strict CArG-box definition as a start compared to the first analysis). This script determines if a motif with a palindromic definition (such as NCC(A/T)_6_GGN) has certain combinations of three nucleotides (henceforth referred to as trimers) on the 3′ side that occur more often than expected by chance. The script finds all occurrences of the palindromic motif in a set of sequences, and counts the occurrences of 5′-motif-trimer-3′ and 5′-reverse complement of trimer-motif-3′ (this is the same when looking in reverse complement). Then, it divides the count by two times the total amount of motif occurrences to give an observed trimer frequency. The expected trimer frequency is then calculated by multiplying the trimer nucleotide frequencies in the given sequences. After this, the relative trimer frequency is calculated using the formula

$$ relativetrime\mathrm{r} frequency=\frac{observedtrimerfrequency}{expectedtrimerfrequency} $$

Next, the input sequences, with the exception of the motif itself, were shuffled and a new analysis of extensions was done. This was done 1,000,000 times and the amount of times that the relative frequency of a certain trimer in the shuffled sequences was higher than the relative trimer frequency in the actual sequences was counted to obtain a *p*-value.

### Motif conservation analysis among *Arabidopsis thaliana* ecotypes

Datasets containing the location of SNPs and 1–3 bp deletions in *Arabdidopsis* ecotypes were downloaded from the 1001 Genomes project website (1001genomes.org) [26, 27]. The datasets used were “MPICao2010”, “Salk” and “MPICWang2013”. To ensure quality of the analysis, only SNPs and small deletions with a quality score of 25 or higher were used in the analysis. A total of 595 ecotypes, including Col-0, were analyzed.

**Fig. 1:**
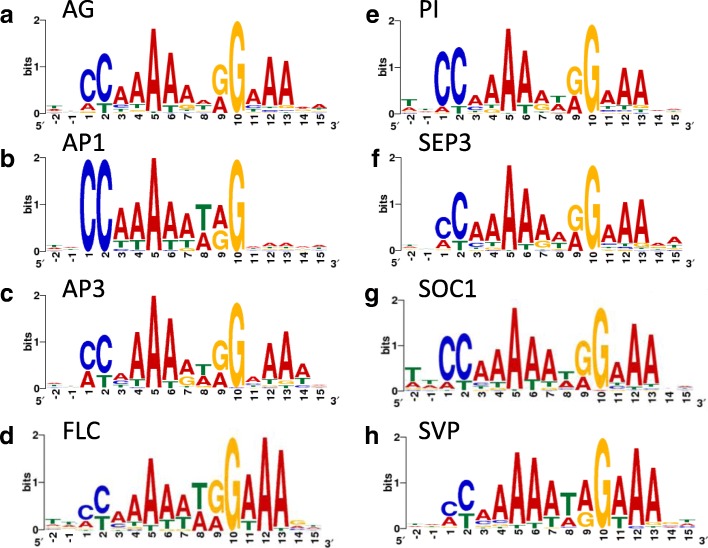
CArG-box like binding motifs for MADS domain proteins involved in flower formation. Logos represents CArG-box motifs found by MEME. **a** AG (**b**) AP1 (**c**) AP3 (**d**) FLC (**e**) PI (**f**) SEP3 (**g**) SOC1 (**h**) SVP

For the conservation analysis, matches to the CArG-box motif of AG (Fig. 1a) were searched in all datasets using FIMO [22], as this motif was representative for all datasets. For each position in each motif occurrence, the relative frequency of each nucleotide in that position in the different ecotypes was calculated. From these frequencies, the Shannon entropy (H) was calculated for each position using the formula H = −∑*p*_*i*_ log_2_ *p*_*i*_, with *p*_*i*_ the relative frequency of a nucleotide among all ecotypes in that position; *i* indexes the four different nucleotides at a given position.

The entropy was averaged per position for all motif occurrences and divided by a background averaged entropy to give a mutation index for each position; this allows to compare entropy of a given motif position with random expected values of entropy. To define the background averaged entropy of a position, which is needed to calculate the mutation index, random positions in ChIP-seq peaks were analyzed for their entropy among *Arabidopsis* ecotypes in a similar way as the analysis of entropy in positions of the motif. Since adenine and thymine have a slower spontaneous mutation rate than cytosine and guanine [28], the exact same amount of the nucleotides that occur in a given position were analyzed as a background. This background was calculated for a total of 10,000 times and the average for each position was used to calculate the mutation index: the average entropy for a given position for all motif occurrences was divided by the average entropy for that position in the background. Furthermore, the amount of times that the background average entropy of a position was higher or lower than the actual average entropy for a position was counted. Positions that had a higher or lower average entropy than 95% or more of the background sets were considered significantly less or more conserved than the background respectively.

To determine if the observed differences in conservation between all CArG-boxes and the subset of perfect CArG-boxes were significant, the mutation index for 10,000 random subsets of 428 CArG-boxes (the same amount as the amount of perfect CArG-boxes) was determined with the average of 25 background sets per subset to calculate mutation frequencies. Positions in perfect CArG-boxes that had a higher or lower mutation index than 95% or more of the 10,000 random sets were considered significantly less or more conserved than the full set of CArG-boxes respectively.

For each position in the CArG box, the correlation between its mutation index (based on conservation in the ecotypes) and the entropy of that position in all matches to the motif in the ChIP-seq peaks (in the Col-0 genome) was calculated. The Shannon entropy of each position was calculated using the formula H = −∑*p*_*i*_ log_2_ *p*_*i*_, where *p*_*i*_ is the relative frequency of each nucleotide *i* at the specific position in Col-0.

## Results

### A CArG-box like motif is enriched in all datasets

Raw data from ChIP-seq experiments for AG [13], AP1 [15], AP3 [16], FLC [10], PI [16], SOC1 [9], SVP [10] and SEP3 [15] were re-analyzed. General dataset characteristics are shown in Table 1. The Pearson correlation between the number of binding sites after re-analysis and the number of binding sites in the original publications was 0.97. This indicates that for most datasets the number of inferred binding sites was relatively similar to those from the original publications. Nevertheless, for two datasets (AG and FLC) there was a somewhat larger difference (Additional file 2: Table S2). In line with this, clustering the datasets based on similarity of peak positions indicated that for all datasets except for AG and FLC, the original peak set and our re-analyzed peakset were most similar to each other (Additional file 3: Figure S1). Overall, these results underline the importance of a uniform re-analysis of the various datasets.

**Table 1:** Summary of the analyzed datasets

Protein	Number of peaks	Amount of reads in sample file	Amount of reads in control file
AGAMOUS (AG)	897	26,754,529	33,740,022
APETALA1 (AP1)	789	33,454,823	47,828,731^a^
APETALA3 (AP3)	1237	31,863,205	29,265,976
FLOWERING LOCUS C (FLC)	59	18,810,650	19,800,993
PISTILLATA (PI)	2156	27,679,860	29,265,976
SEPALLATA3 (SEP3)	4447	40,853,093	47,828,731^a^
SUPPRESSOR OF OVEREXPRESSION OF CONSTANS1 (SOC1)	301	31,448,718	35,116,752
SHORT VEGETATIVE PHASE (SVP)	445	22,114,548	54,952,456

^a^AP1 and SEP3 have the same control file

In order to find enriched motifs in each dataset, de novo motif discovery was carried out using MEME-ChIP [20]. This de novo motif discovery program searches for overrepresented motifs in the central 100 bp of a set of given sequences and compares it to a background, which is made from the nucleotide frequencies in the provided sequences. In all datasets a motif similar to the canonical CArG-box (CC(A/T)_6_GG) was found (Fig. 1a-h; Additional file 4: Table S3).

When describing the motifs in the following sections, each position will be numbered in a way that matches the canonical CArG-box. This canonical CArG-box consists of 10 positions. In the motifs described here the first C that matches the first C from the canonical CArG-box will have position 1 and the last G that matches the last G from the canonical CArG-box will have position 10. Positions before the first C will have negative values, starting at − 1 and positions after the last G will have positive values starting at 11.

Similar and distinct features were found for the CArG-box like motifs in the different datasets. For example, the A/T-core of all motifs consisted mostly of A’s, especially at position 5. Furthermore, all motifs except that from AP1 showed high occurrence of A’s at position 12 and 13. Interestingly, position 9 of the CArG-box was in all motifs almost as often an A as it was a G. Furthermore, the C’s on positions 1 and 2 of the CArG-box often had an A or a T as an alternative, respectively. This effect was strongest for AP3 and weakest for AP1.

Distinguishing features of motifs in individual datasets were the absence of A’s after the motif and the almost 100% occurrence of C’s at positions 1 and 2 in AP1, the relatively high occurrence of the G at position 7 in AP3, the T at position − 2 in PI and SOC1 and the high occurrence of the T at position 8 in AP1, AP3, FLC and SVP. Finally, for FLC and SVP at position 11 the T is a relatively common alternative to the A. Notably, many targets are bound by both FLC and SVP [10], which are able to form heterodimers and hence the obtained motifs are highly similar.

Because the motifs were so similar, it was calculated how many CArG-boxes that contributed to the overall motifs were unique for each MADS TF in the sense that the CArG-box is only present in a peak of a single TF (note that uniqueness refers here to the *position* in the genome; such CArG-box may or may not have unique *sequence*-features). For all datasets except SEP3 and SVP, the vast majority of CArG-boxes of a dataset occurred in one or more other datasets (Additional file 5: Table S4). We also calculated the percentage of unique peaks (meaning that a peak is only present in a dataset of a single TF; see Additional file 6: Table S5). De novo motif discovery in these unique peaks led to the discovery of CArG-box like motifs specific for PI, SEP3 and SOC1. These motifs were similar to the motifs found in the full set, with some minor differences (Additional file 7: Figure S2). The fact that the percentage of specific CArG-boxes bound by all 8 TFs (full overlap) is very low, indicates that there is binding specificity for a particular CArG-box motif by MADS-domain TFs.

### The perfect CArG-box is the most relevant CArG-box variant

Based on the sequence logos the CArG-box like motifs for the different MADS TFs seemed to have many similar features. Therefore, general features of CArG-boxes in all datasets were further examined. First, the relative enrichment of the perfect CArG-box (CC(A/T)_6_GG) and two common variants known from literature (CC(A/T)_7_G and C(A/T)_8_G) in peak centers compared to a background of promoter regions was determined. A peak center was defined as the 250 bp upstream and downstream of a peak summit.

**Fig. 2:**
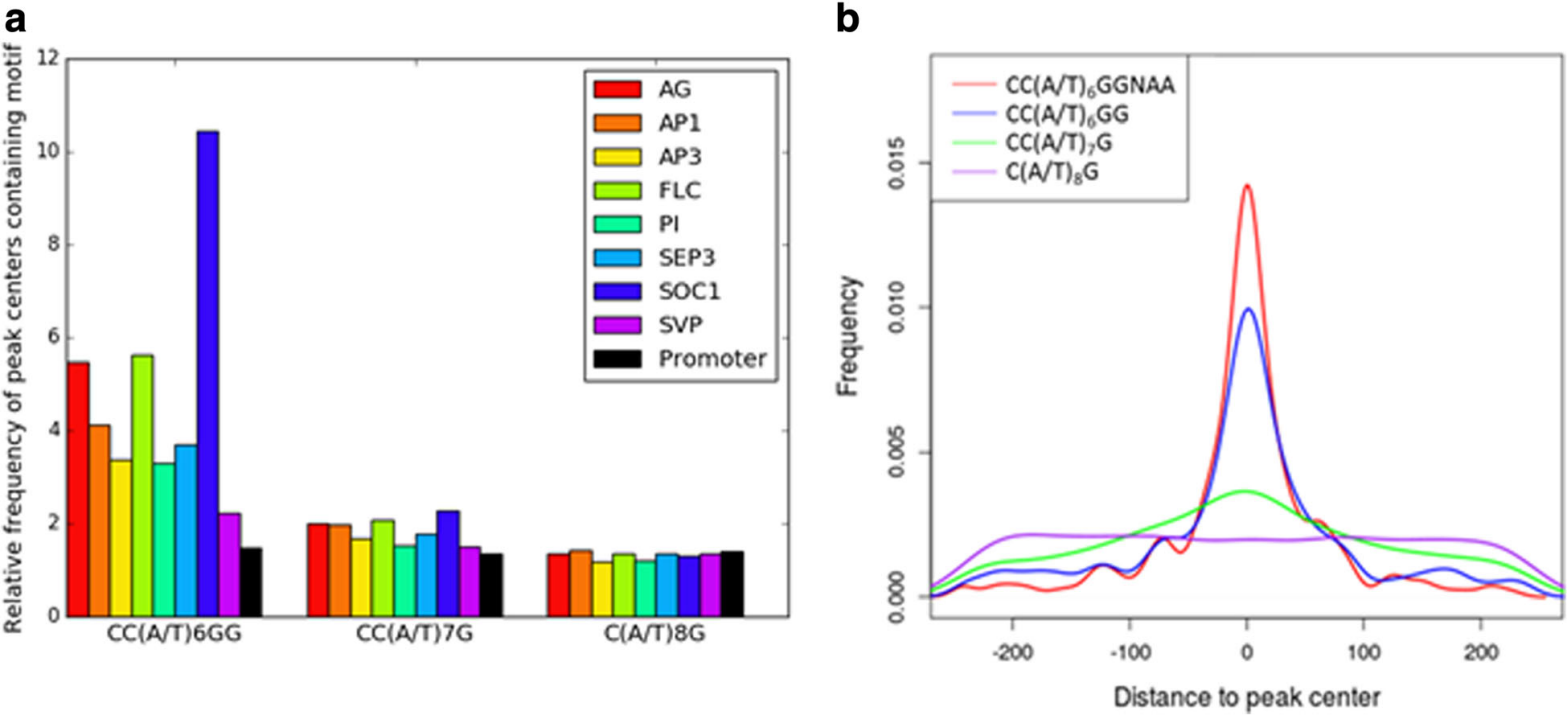
Enrichment of CArG-box variants in peak centers. A peak center is defined as the 250 bp upstream and downstream of the peak summit. **a** Frequency of peak centers containing different CArG-box variants divided by the frequency in random 500 bp stretches in the *Arabidopsis thaliana* genome. Black, relative frequency of CArG-box variant in all *Arabidopsis* promoters. **b** Kernel density plot of positions of different CArG-box variants in peak centers of SEP3 relative to peak summits

The perfect CArG-box was the most enriched, with an enrichment ranging from around 2-fold for SVP, 3-fold for AP3 and PI to over 10-fold for SOC1 (Fig. 2a). Relative enrichment of the variant CC(A/T)_7_G was much lower, ranging from around 1.5-fold for PI to around 2-fold for the other datasets, except for SVP (Fig. 2a). In all datasets, the variant C(A/T)_8_G was not enriched compared to the promoter background (Fig. 2a).

In ChIP-seq data it is expected that the protein of interest binds to the DNA directly underneath a peak summit. Therefore, most occurrences of a directly bound motif are expected to be close to the peak summit, whereas an unbound motif is expected to be evenly distributed throughout the peak. Relevant motifs are therefore expected to be centrally enriched. To determine the relevance of different variants of the CArG-box, their central enrichment was determined in the different datasets. Figure 2b shows central enrichment of motif variants for SEP3. Central enrichment of motifs in the other protein datasets show the same general trend as motifs in the SEP3 dataset (Additional file 8: Figure S3).

In all datasets the perfect CArG-box was the most centrally enriched motif of the three CArG-box variants analyzed. The variant CC(A/T)_7_G was much less centrally enriched and the motif variant C(A/T)_8_ was not centrally enriched at all.

Because in the sequence logos the A’s at position 12 and 13 seem to be important, central enrichment of a perfect CArG-box with the extension -NAA was tested. This CArG-box variant, CC(A/T)_6_GGNAA was even more centrally enriched than the perfect CArG-box without the extension (Fig. 2b). Central enrichment of the CC(A/T)_7_G motif was also higher with the 5’-NAA-3′ extension, although still less than the perfect CArG-box. In contrast, the motif C(A/T)_8_GNAA was still not centrally enriched (data not shown).

To ensure that the results on motif enrichment and central enrichment were not biased by the presence of weakly bound peaks, the analysis was repeated using only the top 500 peaks for each MADS protein (except for FLC, SOC1 and SVP, which only had 59, 301 and 445 peaks respectively and were left the same for this analysis). Results obtained using this analysis were qualitatively similar to the results reported above. In addition, we checked whether the results would be artificially influenced by the fact that some of the tested motifs are more degenerate than others. As a negative control, the motif CC(A/T)_6_GGNTT was used instead of CC(A/T)_6_GGNAA, and the motif CC(A/T)_6_CG instead of CC(A/T)_6_GG; both negative controls were indeed much less centrally enriched than the motifs themselves. Finally, we tested if central enrichment may be influenced by the fact that more degenerate motifs occur more often multiple times in a given peak region. We found that the variants CC(A/T)_7_G and C(A/T)_8_G indeed occur more often together in a peak than the canonical motif CC(A/T)_6_GG. However, the vast majority of CArG-box variants still only occur once within a peak center (Additional file 9: Table S6, panel A). Therefore, it is unlikely that this influences the analysis of central enrichment. Similarly, there was no strong trend when comparing co-occurrence of combinations of different CArG-box variants in peaks (Additional file 9: Table S6, panels B-D); co-occurrence was always about as frequent as would be expected from the frequencies of peaks that contained each motif variant.

### Other motifs are also enriched in the datasets

De novo motif discovery using MEME-ChIP also resulted in the discovery of other enriched motifs (summarized in Table 2). In all datasets except FLC and SVP, a motif consisting almost entirely of A’s and G’s was enriched (Additional file 10: Figure S4). When comparing with the promoter background, this motif was hardly enriched (less than 1.5 fold); it was also not centrally enriched and therefore, most likely not relevant for MADS domain TF binding. Three motifs known from literature [17, 29] were also enriched in some of the datasets: motifs similar to the G-box were found in all datasets except in AP1, FLC and SOC1 (Additional file 11: Figure S5), whereas a motif similar to the motif for TCP type II was found in AP1 and SOC1 and motifs for TCP class I and class II were found in the SEP3 dataset (Additional file 12: Figure S6).

**Table 2:** Summary of secondary motifs found^a^

Dataset	GA/CT-rich motif	G-box	TCP type I motif	TCP type II motif	WRKY-like motif
AG	Yes	Yes	No	No	No
AP1	Yes	No	No	Yes	No
AP3	Yes	Yes	No	No	No
FLC	No	No	No	No	No
PI	Yes	Yes	No	No	No
SEP3	Yes	Yes	Yes	Yes	Yes
SOC1	Yes	No	No	Yes	No
SVP	No	Yes	No	No	No

^a^ Sequence logos of the motifs summarized in this table can be found in Additional file 10: Figure S4 (GA/CT-rich motif), Additional file 11: Figure S5 (G-box) and Additional file 12: Figure S6 (TCP type I and II). The WRKY-like motif is defined as GTTGACTTT

**Fig. 3:**
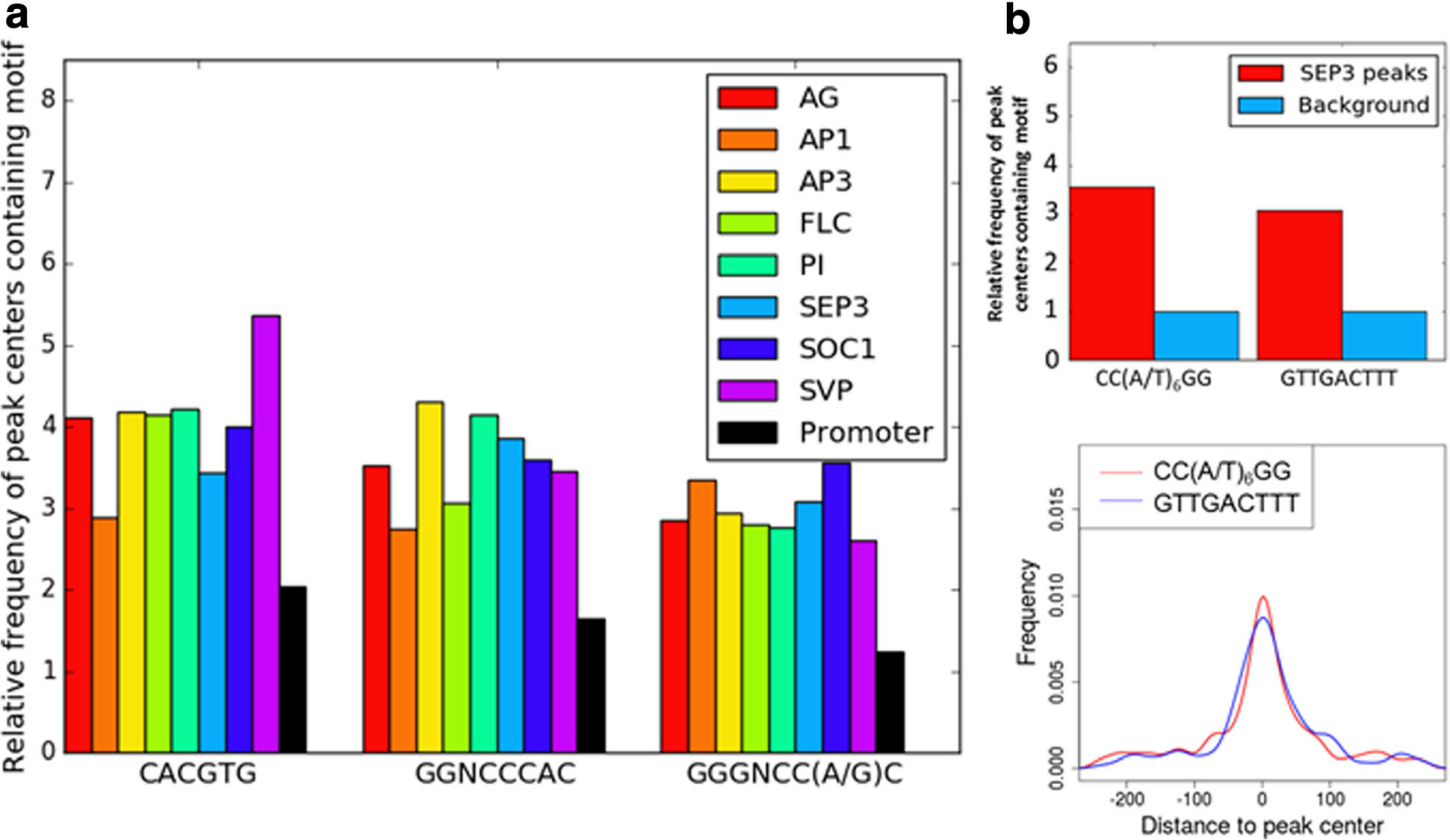
Non-CArG box motifs. **a** Relative enrichment of peak centers containing a secondary motif compared to the promoter background. The frequency of peak centers containing a secondary motif was calculated and divided by a background frequency. A peak center is defined as the 250 bp upstream and downstream of a peak summit. G-box: CACGTG, TCP class I: GGNCCCAC, TCP class II: GGGNCC(A/G)C. **b** Enrichment of a WRKY-like motif (GTTGACTTT) in SEP3 peaks. **c** Kernel density plot of positions of the perfect CArG-box and the WRKY-like motif in the peak center compared to the peak summit

MEME-ChIP may overlook weaker motifs that are not strongly enriched compared to nucleotide frequencies used as background model, but that are still enriched compared to the frequency the motif has in the rest of the genome. To overcome this problem, analysis of overrepresentation of the G-box and the two TCP-motifs in peak centers compared to the promoter background was also carried out. All three motifs were enriched in peak centers compared to the background in all datasets (Fig. 3). In all datasets, the three motifs showed enrichment towards the peak center, although this enrichment was less strong than for the CArG boxes (Additional file 13: Figure S7). All motifs were about equally centrally enriched in the datasets. No clear correlation between the occurrences of CArG-boxes and other motifs in a peak could be found.

Above, motifs were found by analyzing enrichment compared to a background, followed by analysis of central enrichment. To analyze central enrichment on its own, a custom Python script aimed at finding centrally enriched motifs was written. With this script, a new motif (GTTGACTTT) that was low in abundance (89 cases in 4447 peaks) but almost as centrally enriched as the perfect CArG-box was found in the SEP3 dataset (Fig. 3b,c). Relative enrichment of this motif in ChIP-seq peaks of SEP3 was also comparable to that of the perfect CArG-box (Fig. 3b,c). The motif occurred in only 48 cases in the same peak as a CArG-box, which is 25% less than expected by chance. This motif is similar to the W-box motif of WRKY transcription factors, which has the consensus TTGACC/T [30].

### Individual CArG-box sequences in binding sites of different MADS proteins

As indicated above, a perfect CArG-box (CC(A/T)_6_GG) was strongly enriched for each of the different MADS TFs. In order to further investigate potential differences between binding sites for different MADS TFs, the individual sequences confering to the perfect CArG-box that occured in the binding sites were analysed. When focussing on the CArG-box sequence itself, the most striking pattern was a preference for all the MADS TFs for consecutive stretches of A. In particular, the different perfect CArG-box sequences containing at least three consecutive A nucleotides accounted for 79% (for SVP) - 94% (for FLC) of the individual sequences. These numbers are much higher than would be expected randomly: out of all different perfect CArG-box variants, 20 out of 36 i.e. 56% contain at least three consecutive As. When requiring an even more specific variant with at least four consecutive A nucleotides, still 45% (SVP) – 61% (SOC1) of the individual sequences contained such AAAA stretch. Here the difference with random expectation is even larger: out of all different perfect CArG-box variants, only 8 out of 36 i.e. 22% contain at least four consecutive As.

The analysis of individual sequences within the perfect CArG-box itself did not indicate clear differences between the different MADS TFs. When performing a similar analysis on the 3′-extension of perfect CArG-boxes, some differentiation between the different TFs was obtained. As indicated in Additional file 14: Figure S8, whereas a large variety of sequences occured for SEP3, for the other MADS TFs much more restricted sets were observed. Clustering the TFs based on their preferred extension-sequences recovered relevant pairs of MADS TFs: PI and AP3 were clustered together, as were FLC and SVP (Additional file 14: Figure S8). However, in almost all cases the sequences that occur both for PI and AP3 or both for FLC and SVP are cases where the same binding site occurs for these two TFs. In addition to these differences between MADS TFs, the analysis of individual sequences again indicated a clear preference for the NAA extension (Additional file 14: Figure S8).

### Overrepresentation of CArG-box extensions suggests the existence of a hybrid CArG-box - TCP binding site

To further investigate the nature of the 3′ extension of CArG-boxes, the overrepresentation of specific extensions was analyzed. First, the nucleotides directly adjacent to CArG-box like sequences defined by MEME-ChIP were investigated. We denote the positions of these nucleotides using 5′ and 3′ with respect to the CArG-box orientation as defiend by MEME-ChIP, which is oriented to align as much adenines as possible in the CArG-box core. The occurrences of different sequences of three nucleotides at the 5′ side of position 1 (from hereon 5′ extensions) and the 3′ side of position 10 (from hereon 3′ extensions) and combinations of 5′ and 3′ extensions were counted. A striking pattern could be seen (Additional file 15: Table S7). Whereas on the 3′ side there was clear preference for the extension 5′-AAA-3′ followed by variants of 5’-NAA-3′, the preference for certain sequences on the 5′ side was much weaker. Generally, the sequences 5′-AAA-3′ and 5′-TTT-3′ occurred most often.

**Fig. 4:**
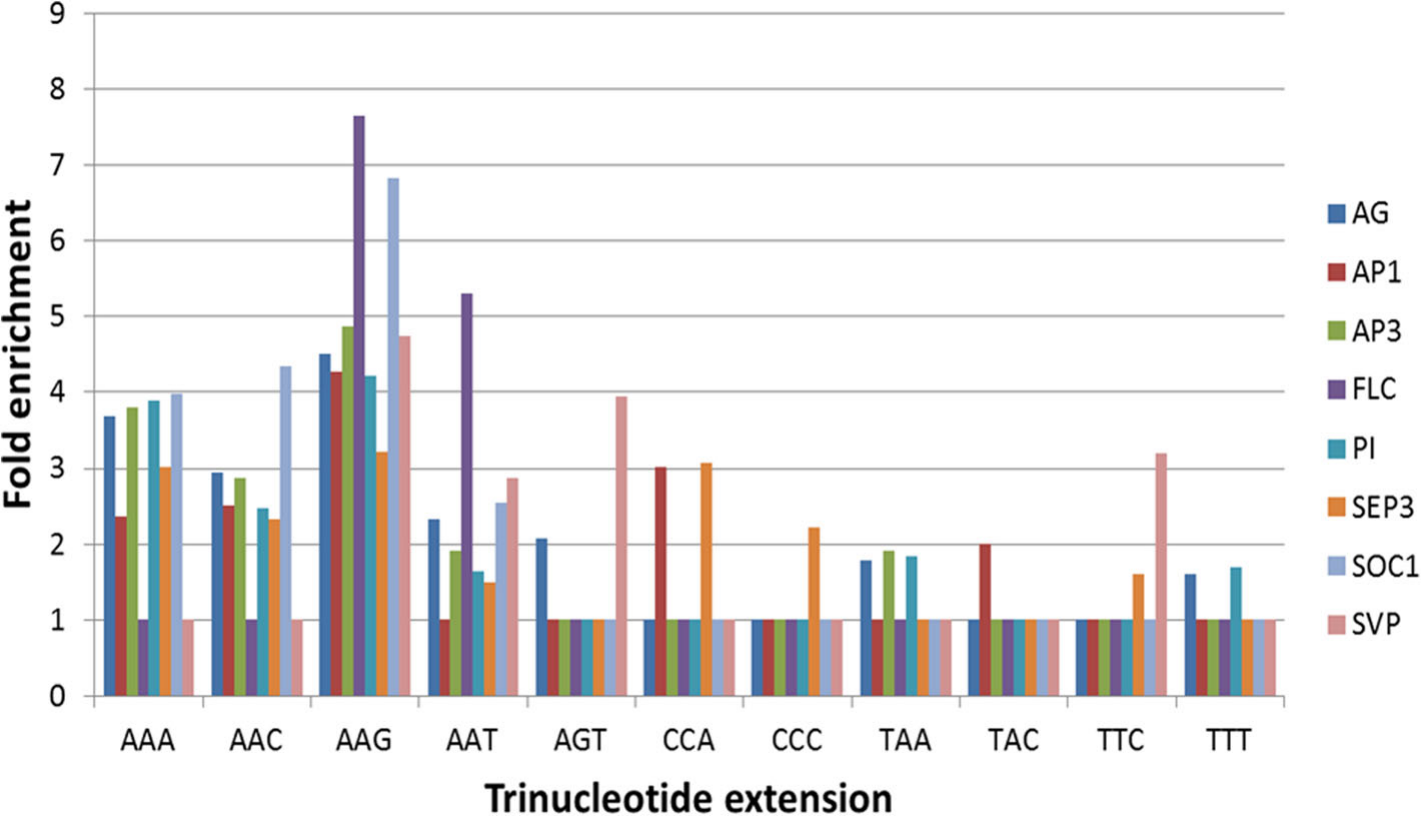
Significantly overrepresented 3′ extensions of the CArG-box core. Enrichment of extensions of 3 nucleotides is calculated as the frequency of the extension after the CC(A/T)_6_GGN-core in ChIP-seq peaks divided by the expected frequency of the extension based on the frequencies of nucleotides in the ChIP-seq peaks. All extensions are depicted for which at least one dataset is significant at *p* < 0.05. For visualization purposes, all extensions that are enriched relative to what is expected from nucleotide frequencies, but are not significant, are set to 1. Note that a similar analysis, but for CArG-box like sequences picked up by MEME-ChIP, is presented in Additional file 15: Table S7

Next, we focused on nucleotides on the 3′ side of manually defined perfect CArG-boxes. For each of the datasets the frequency of all combinations of three nucleotides after occurrences of CC(A/T)_6_GGN in ChIP-seq peaks was counted and divided by an expected frequency given the nucleotide distribution of the ChIP-seq peaks analyzed. Statistical significance was assessed by comparison with random permutations (Methods). In all datasets, different combinations with A’s were significantly enriched (Fig. 4). This is in line with the importance of the A-containing extension discussed above. In the dataset of SEP3 however, two additional combinations of nucleotides were significantly enriched, namely 5’-CCC-3′ and 5’-CCA-3′ (Fig. 4). Adding these extensions to the CArG-box core used in this analysis gives the motif CC(A/T)_6_GGNCCC or CC(A/T)_6_GGNCCA, which is a combination of the CArG-box and the core of the class I (GGNCCC) and class II (GGNCCA) TCP binding motif, respectively. It has to be noted however that the occurrence is only 31 for the extension CCA and 11 for extension CCC, but due to the low frequency of C’s in ChIP-seq peaks this is still a significant enrichment.

In addition to the combined CArG-box – TCP motif, when analyzing TCP motifs in the ChIP-seq peaks a more general trend in position of the TCP motif vs. the CArG-box was observed, in particular for perfect CArG-boxes. Compared to all CArG-boxes (perfect and non-perfect) in MADS ChIP-seq peaks, there was a slight preference for TCP motifs to be positioned 3′ to CArG-boxes (261× TCP motif 3′ to CArG-box vs. 217× TCP motif 5′ to CArG-box). However, when using only perfect CArG-boxes, this preference was much stronger, with 68 cases with the TCP motif 3′ to the CArG-box, and only two cases with the TCP motif 5′ to the CArG-box. Note that only in the case of a perfect CArG-box a TCP motif can be formed 3′ to the CArG-box as a hybrid MADS-TCP binding site.

### Perfect CArG-boxes are better conserved than the full set of CArG-boxes

Conservation of CArG-boxes within 595 *Arabidopsis* ecotypes was evaluated to investigate the evolutionary importance of CArG-boxes. Almost two third of the CArG-boxes analyzed (obtained from the Col-0 ChIP-seq data) had at least one mutation in any of the 595 ecotypes (Additional file 16: Table S8; Additional file 17: Figure S9, panel A). A mutation index was calculated for each position from all the CArG-boxes using all ecotypes. In short, this mutation index indicates the mutational variability of that position between all ecotypes compared to a background mutational variability in ChIP-seq peaks, taking into account that some nucleotides mutate faster than others. This mutation index was calculated by dividing the average entropy for a given position in the motif by an average background entropy, calculated specifically for each position (Methods). A mutation index of 1 indicates that a position is as conserved as the background, whereas a lower mutation index indicates that the position is more conserved than the background.

**Fig. 5:**
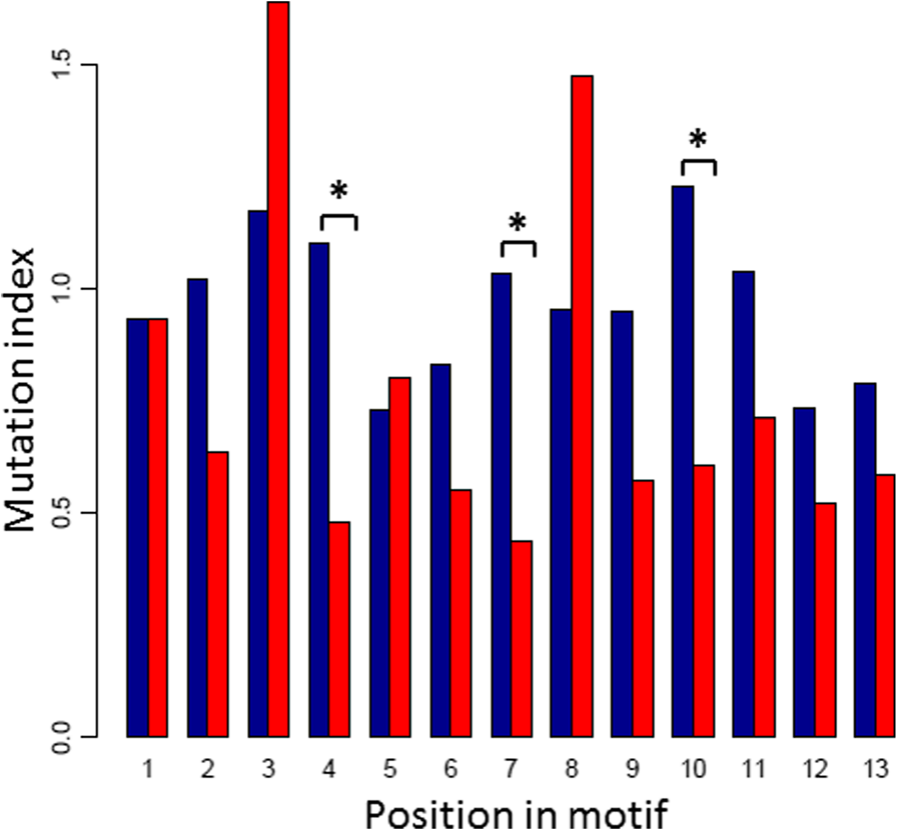
Conservation of CArG-boxes in ChIP-seq peaks among *Arabidopsis thaliana* ecotypes. For each position in each CArG-box, mutational entropy was divided by an average background entropy to give a mutation index (blue), averaged over all motif occurrences. This was also done for the subset of 428 perfect CArG-boxes (red). Positions for which the difference in mutation index between perfect CArG-boxes and all CArG boxes was statistically significant are indicated with an asterisk

For the full set of CArG-boxes the overall average mutation index was 0.96 (+/− 0.15). Positions that were relatively well-conserved were position 5, 6, 12 and 13, whereas the most variable positions when analyzing ecotypes were position 3 and 10 (Fig. 5). In general terms, the trend was that positions that were less variable in the motif logo based on matches in ChIP-seq peaks (Fig. 1) also were less variable between *Arabidopsis* ecotypes, as indicated by a lower mutation index, with the notable exception of position 10 (Additional file 17: Figure S9, panel B). In other words, although position 10 is quite strongly defined as “G” in the CArG-box motif (Fig. 1) it is relatively less conserved between ecotypes. The mutations observed for this “G” in the ecotypes showed a distinct trend. Of the 522 unique changes (occurring in one or more ecotypes), more than half (282) were to an A. Furthermore, 54 were to a C, 120 to a T and there were 66 deletions. In all these cases, the CArG-box would be destroyed based on the logo shown in Fig. 1.

Interestingly, when subsampling only the perfect CArG-boxes from the full set, the average mutation index was substantially lower, 0.76 (+/− 0.36). This means that the perfect CArG-boxes are on average much better conserved than the non-perfect CArG-boxes. All positions in perfect CArG-boxes, except positions 3 and 8, had a mutation index below 1, i.e. they were more conserved than the background (Fig. 5). Important to note is that position 10, which is not conserved when all CArG-boxes are taken into account, appears highly conserved in perfect CArG-boxes (mutation index 0.61).

To analyze the significance of the observed difference between perfect and non-perfect CArG-boxes, 10,000 random subsamples were taken from the full set of CArG-boxes and the mutation index was calculated for each position in each subsample. For positions 4, 7 and 10 it was found that in 95% or more of the cases the mutation index was lower in the subsample of perfect CArG-boxes than in the random subsamples, suggesting that the observed difference in mutation index between the subset of perfect CArG-boxes and the full set was significant (indicated by an asterisk in Fig. 5). With a similar analysis it was found that the observed higher mutation indices of positions 3 and 8 of the subsample of perfect CArG-boxes were not significant.

## Discussion

Previous research on MADS-domain proteins has indicated that the preferred DNA binding motif of this protein class is the CArG-box, which has the consensus CC(A/T)_6_GG. Several studies using ChIP-seq with different proteins have revealed particular characteristics of the CArG-box in *Arabidopsis*. However, due to different analysis methods a comparison of similarities and differences in preferred binding motifs of MADS-domain proteins based on data described in these articles is not possible. In the present study, eight ChIP-seq datasets from MADS-domain proteins that regulate the floral transition and flower development in *Arabidopsis thaliana* were re-analyzed. Similar and distinct features of the preferred binding motifs of the proteins were pinpointed and the importance of CArG-boxes was examined from an evolutionary perspective by looking at conservation of CArG-boxes within *Arabidopsis thaliana* ecotypes.

Using the de novo motif discovery tool MEME-ChIP, highly similar CArG-box like motifs were found in all datasets. This could be explained by the fact that MADS-domain proteins predominantly bind DNA as heterodimers or (hetero-) tetramers and that a MADS-domain protein can form different heterodimers (except for the obligate AP3-PI heterodimer) in a particular ChIP sample. Therefore the binding motif actually represents an ‘average’ motif that is composed of the motifs for multiple combinations of heterodimers. With this analysis, motifs specific for a protein may become masked by the overwhelming amount of other sequences that are less specific for the protein, or even not detected at all. An alternative interpretation that we cannot completely rule out would be that the different CArG-box like motifs, although highly similar, contain sufficient variation to discriminate at least to some extent the different MADS TFs. Further studies using e.g. a more predictive computational approach instead of a descriptive motif search might shed more light on this.

Next, we investigated how many CArG-boxes are unique to a specific dataset (meaning the position where they occur is only part of a peak in that particular dataset). Note that such CArG-boxes may or may not have unique sequence-features. With the exception of SEP3 and SVP, this number was very low compared to the total amount of CArG-boxes, and even the number of CArG-boxes that overlapped with only one dataset was relatively low. This suggests that many CArG-boxes are not specific for a certain protein, but can also be bound by other MADS-domain complexes. Nevertheless, small variations in CArG-boxes and extensions were found among the different MADS-domain TFs. Whether these preferences reflect selective binding and hence specificity in target gene regulation remains to be seen.

To find additional CArG-box like sequences specific for a dataset, we carried out de novo motif discovery in peaks that were unique for each dataset. We speculated that in this way we might not only find CArG-boxes that were already found but perhaps also sequences that looked like a CArG-box but that were not considered part of the motif when all peaks of a dataset were used for the analysis. However, a CArG-box like motif in these unique peaks was found only for PI, SEP3 and SOC1. CArG-boxes in unique peaks of PI, SEP3 and SOC1 formed motifs that were slightly different from the motifs from all peaks of the respective datasets. This suggests that a portion of these peaks is caused by binding of the protein to a CArG-box that is specific for the protein.

**Fig. 6:**
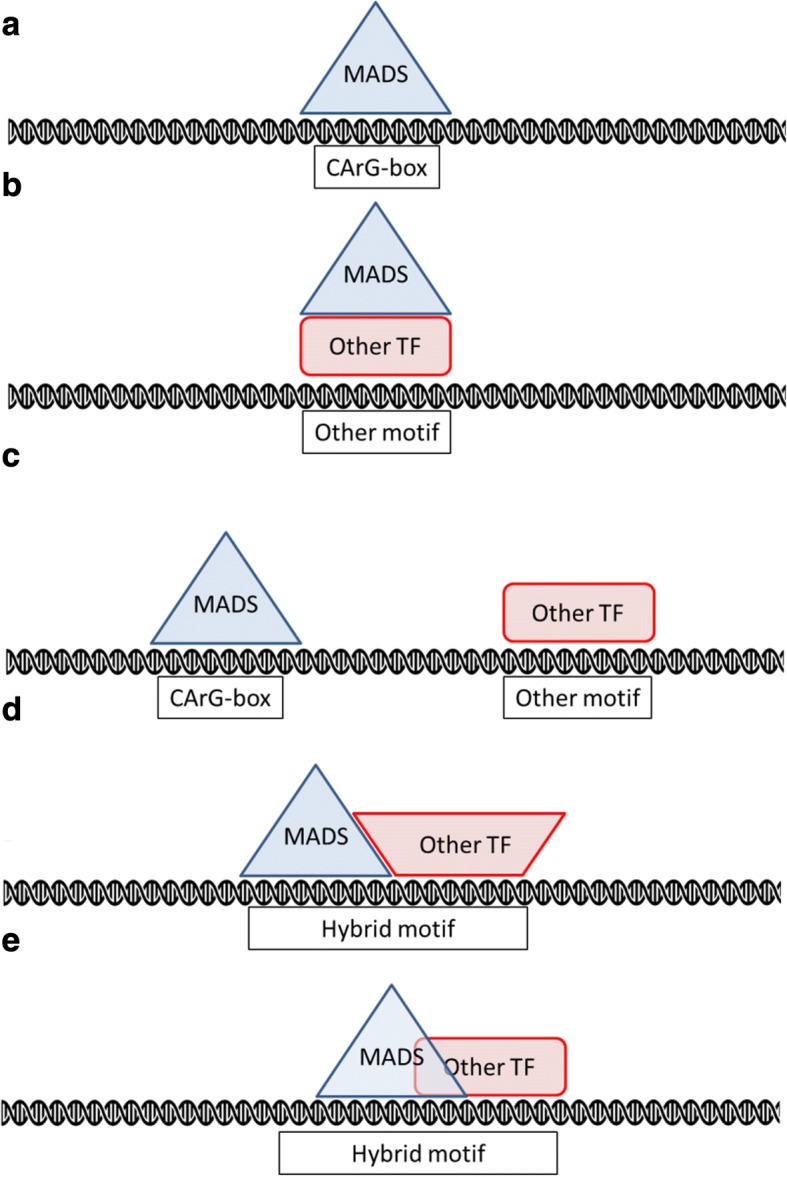
Five models that explain the occurrence of different binding motifs in MADS-domain protein ChIP-seq data analyzed in the present study. **a** The MADS-domain protein binds to a CArG-box. **b** The MADS-domain protein binds to another transcription factor, which binds DNA at a motif specific for that transcription factor. **c** Same as (**a**), but because by chance or as part of an enhanceosome there is a binding site of another transcription factor close by, both the CArG-box and the other motif occur in the ChIP-seq peak. **d** The MADS-domain protein needs another transcription factor for binding to a motif that is a hybrid between a CArG-box and the motif for the other transcription factor. **e** The motif is competitively bound by the MADS-domain protein and another protein and is therefore a hybrid between a CArG-box and the motif of the other transcription factor

In summary, the general motif of the CArG-box is rather similar for the different datasets analyzed. This raises the question whether there are other properties than the CArG-box sequence that enable MADS-domain proteins to bind to specific genomic sequences and regulate different physiological and developmental processes. Of course, the limited variation in the motifs of the different proteins could be due to limitations of the Position Weight Matrix (PWM) motif model used by MEME. For example, the PWM does not take dependencies into account, e.g. is the base at position 2 dependent on the base at position 9 in the CArG-box motif. Other explanations are however possible as well (Fig. 6). A possible scenario is that different proteins bind to the same sequence, but regulate the same genes differentially [12]. Another possibility is that they interact with certain co-factors or other TFs that facilitates cooperativity, which might be important for specificity (Fig. 6b and d). The identification of several other motifs in the present study supports this hypothesis, although the motifs found in the different datasets were generally quite similar. Furthermore, the spacing between CArG-boxes could also be important since MADS-domain TFs are able to bind as tetramers to two adjacent CArG-boxes as proposed in the Quartet-model [31]. When a tetramer is binding to two CArG-boxes, it is possible that cooperativity facilitates the TF binding to ‘weak’ binding sites, which will blur the overall motif.

Another aspect that may explain binding of MADS-domain proteins to specific stretches of DNA are the structural properties of the DNA. It was recently demonstrated that taking DNA shape descriptors into account the predictability of DNA-binding sites for various transcription factors, including MADS domain TFs could be improved [32]. More specifically, we previously noticed that the A/T-core of CArG-boxes in SEP3 binding peaks resembles a structural motif called the A-tract [33], a motif associated with DNA bending towards the minor groove [34]. The degree of DNA bending resulting from a TF binding to the A-tract was hypothesized to contribute to specificity. In the present study we confirmed the presence of consecutive stretches of A in the binding sites of the various MADS TFs that we analyzed. Furthermore, the sequence at the 3′ side of the canonical CArG-box also consists mostly of A’s. Note that obviously for A one can also read T here, depending on the strand; the point is that the core of the CArG-box, and the extension, is not a random combination of A’s and T’s.

Because CArG-boxes were highly similar between datasets, we next focused on similar aspects of CArG-boxes in all datasets. Based on relative and central enrichment, the perfect CArG-box appeared to be the most important motif of the three general CArG-box variants described in literature [11, 12]. The fact that the variant C(A/T)_8_G is barely relatively enriched and not centrally enriched indicates that this motif is not important for binding of the MADS-domain proteins analyzed in this study, even though it cannot be ruled out that some of the sequences belonging to this motif are still bound by these proteins.

In the eight datasets studied, central enrichment of the perfect CArG-box with the extension NAA as well as the variant CC(A/T)_7_G with the extension NAA was higher than that of each of these core motifs alone. Also, the extension NAA occurs in a large proportion of CArG-boxes found by MEME-ChIP in all datasets. This underlines the importance of these adenines at position 12 and 13 and makes a strong case for extending the consensus motif of a perfect CArG-box to CC(A/T)_6_GGNAA. Additionally, it is striking to see that there is preference for a one-sided extension. This is especially interesting considering the fact that MADS-box proteins bind as dimers and therefore are expected to bind a more or less palindromic motif. The notion that a CArG-box is not actually palindromic is supported by the fact that the A/T core mostly consists of A’s and not a random combination of A’s and T’s, as discussed before. Possible explanations for this are that this kind of non-palindromic motifs lead to a DNA structure that promotes MADS-box protein binding (as discussed above), or that MADS-box proteins often bind as heterodimers and that each side of the CArG-box is optimized for one of the two binding partners.

Interestingly, according to an analysis of overrepresented 3′ extensions of the perfect CArG-box in SEP3, the extensions 5’-NCCC-3′ and 5’-NCCA-3′ were overrepresented. A motif with such an extension contains, apart from a perfect CArG-box, the sequence GGNCCC or GGNCCA, which are the cores of TCP class I and class II motifs, respectively [29]. An intriguing hypothesis is that these motifs are hybrid binding sites between SEP3 and a TCP protein (Fig. 6d). Although it has not been tested if such a hybrid complex could physically bind two stretches of DNA so close to each other, it has been shown recently that two transcription factors can bind to a ‘hybrid’ motif in vitro, a model known as latent specificity [35]. In this model, the binding may be cooperative, requiring both SEP3 and a TCP protein for optimal binding. Another explanation is that this motif is a site of competition between a TCP protein and SEP3 for binding to the promoter (Fig. 6e). In this case, the two proteins are expected to regulate the target gene differentially.

Apart from these hybrid sites, four different motifs from known transcription factor families other than the MADS family were found. These were the G-box, which is bound by bHLH and bZIP proteins [36, 37], the motifs for TCP class I and class II proteins [29] and a motif resembling that for WRKY proteins [38]. A hypothetical explanatory model for this is that the MADS-domain protein interacts with proteins of another transcription factor class, which bind to specific DNA sequences independent of a CArG-box motif. As recently reviewed, such interactions between transcription factors of different families are increasingly recognized to be important [39]. When the MADS-domain protein was immunoprecipitated, the complex with the other TF and the DNA was also precipitated, eventually resulting in read enrichment of the other motif (Fig. 6b). Alternatively, the motifs could also be enriched, because they appear in the same promoter regions where CArG-boxes are located and form a so-called enhanceosome with a defined set of multiple TF binding sites [39] (Fig. 6c). The central enrichment of all motifs suggests that at least some of the motifs are found because they were immunoprecipitated together with a MADS-domain protein, as explained in model 6b (interactions between MADS-domain proteins and other TFs). Again, the WRKY-like motif stands out, as it is much more centrally enriched than the other motifs. This suggests that the occurrence of the WRKY-like motif in the SEP3 dataset is mainly explained by MADS-WRKY interactions. A recent analysis of *Arabidopsis* ChIP-seq data [40] also analyzed co-binding of multiple transcription factors; specifically, G-boxes were observed as relevant for MADS-domain proteins, but TCP binding sites and WRKY motifs were not found in that study.

Interestingly, although interactions between MADS-domain proteins and TCP proteins or bHLH proteins have been described in several organisms [39, 41, 42], to the authors knowledge interactions between MADS-domain proteins and WRKY transcription factors have not been described. However, WRKY transcription factors have been linked to flowering time and growth [43–46], indicating that MADS-WRKY interactions may be relevant for flower development.

Apart from motifs of known transcription factors, in each dataset a motif of variable length was found that consisted mostly of G’s and A’s. It is very likely that this motif is part of a so-called GA element, a promoter element that has been described as an alternative to the TATA-box [47]. Because MADS-domain proteins predominantly bind to promoter regions, it makes sense that this element is often enriched in ChIP-seq peaks of MADS-domain proteins when compared to a genomic background. The fact that this motif is barely enriched when comparing with a promoter background and also is not centrally enriched is in line with the hypothesis that this element occurs in the data only because it is situated close to a binding site, but is not directly or indirectly bound by MADS-domain proteins.

To get a better idea of the evolutionary importance of CArG-boxes and the different nucleotides within a CArG-box, we investigated conservation of CArG-boxes among different *Arabidopsis* ecotypes. Surprisingly, when considering all the CArG-boxes the majority of positions were not better conserved than random positions in ChIP-seq peaks. This includes the C’s of position 1 and 2 and the G’s of position 9 and 10. However, when only perfect CArG-boxes were analyzed, most positions were more conserved compared to the background. This suggests that this CArG-box type is generally more important for MADS-domain protein binding than non-perfect CArG-boxes.

There are several explanations for the fact that CArG-boxes are generally not much better conserved than the background. First of all, the nature of the mutations was not taken into account. This means that there can be non-detrimental mutations. In the future it would be interesting to see if some mutations happen more often than others. It would also be interesting to study if genes that encode important regulatory proteins only have CArG-boxes with non-detrimental mutations in their promoters. Another explanation for the fact that CArG-boxes are not well conserved could be that a mutation within a CArG-box is compensated by another mutation within the CArG-box. This was not considered in the present study. Finally, since many promoters contain multiple CArG-boxes it is possible that other CArG-boxes take over the role of a mutated CArG-box and maintaining the regulation of the corresponding gene. In any case, the more conserved nature of perfect CArG-boxes underlines their importance.

## Conclusions

In this paper, several aspects of DNA binding by eight different MADS-domain proteins were analyzed by re-analyzing ChIP-seq data. MADS-domain proteins bind to a DNA motif called the CArG-box. Based on the presented analysis, it can be concluded that the exact definition of the CArG-box is more flexible than most previous papers suggest. The exact sequence is often different from the classical definition, CC(A/T)_6_GG. Moreover, most positions are almost as often mutated as other nucleotides within the same DNA regions in *Arabidopsis thaliana* ecotypes, especially when the CArG-box does not comply with the classical definition. However, some general features of the CArG-box seem to be important for binding by MADS-domain transcription factors. For example, CArG-boxes that comply with the canonical definition of a CArG-box are more relatively and centrally enriched and better conserved than other CArG-box like motifs. Furthermore, CArG-boxes with the 3′ extension 5’-NAA-3′ are more centrally enriched than CArG-boxes without this extension. In all datasets several motifs of other known transcription factor families were also enriched, suggesting the importance of interfamily transcription factor interactions. The CArG-box motifs found to be enriched in the different MADS-domain ChIP-seq datasets are similar, but not identical for each MADS-domain TF. Whether these small differences are sufficient to explain the proposed specificity of target gene regulation remain to be determined. To be more conclusive about specificity and how transcription factors regulate specific processes, the ChIP data should be complemented with more quantitative DNA binding data (affinities), more information about the composition of the TF complexes including co-factors, and the role of cooperativity in DNA binding and gene regulation.

## Additional files

Additional file 1: **Table S1.** Percentage of peak centers of which at least half the peak center falls within the promoter. (PDF 45 kb)

Additional file 2: **Table S2.** Number of peaks in original analysis and after re-analysis. (PDF 45 kb)

Additional file 3: **Figure S1.** Clustering of original peak sets and peaks sets after re-analysis. Hierarchical clustering with complete linkage (function hclust in R) was performed using as distance measure between two peak sets the average distance between peaks in one set and in the other. Peaks-sets after re-analysis are indicated with name of the transcription factor, original peak sets are indicated with the suffix “orig”. (PDF 38 kb)

Additional file 4: **Table S3.** Percentage of peaks in which motifs occur. For the CArG-box motifs, in addition to the percentage observed in all peaks, the percentage observed in the top 500 peaks is listed as well (between brackets). (PDF 55 kb)

Additional file 5: **Table S4.** Unique and overlapping CArG-boxes in each dataset. CArG-boxes are defined as all non-overlapping CArG-box like motifs found for each of the eight proteins (see Fig. 1 for corresponding motifs). Note that the amount of CArG-boxes occurring in all datasets is not the same for each dataset. This is caused by the fact that that the CArG-box definition obtained by the de novo motif search differs between the datasets. (PDF 50 kb)

Additional file 6: **Table S5.** Unique and overlapping peak centers in each dataset. A peak center is defined as the region 250 bp upstream and downstream of a peak summit. (PDF 52 kb)

Additional file 7: **Figure S2.** CArG-box like motifs in unique peaks in PI, SEP3 and SOC1 datasets. Logo representation of all matches to the motif found by MEME. Logos are from (A) PI (B) SEP3 (C) SOC1. (PDF 126 kb)

Additional file 8: **Figure S3.** Central enrichment of different CArG-box variants in different protein datasets. Kernel density plot of matches to the motif in peak centers relative to the peak summit. Plots are from (A) AG (B) AP1 (C) AP3 (D) FLC (E) PI (F) SEP3 (G) SOC1 (H) SVP. (PDF 251 kb)

Additional file 9: **Table S6.** Co-occurrence of several CArG-box variants in ChIP-seq peaks. (A) Amount of peaks with a single occurrence and with multiple occurrences of pre-defined CArG-box variants. (B) Amount of peaks that contain only CC(A/T)_6_GG, only CC(A/T)_7_G or both motifs. (C) Amount of peaks that contain only CC(A/T)_7_G, only C(A/T)_8_G or both motifs. (D) Amount of peaks that contain only CC(A/T)_6_GG, only C(A/T)_8_G or both motifs. Expected values for B, C and D were calculated by multiplying the frequencies of peaks with each motif and multiplying that with the total amount of peaks of a dataset. (PDF 71 kb)

Additional file 10: **Figure S4.** GA/CT-rich motif. Logo representation of all matches to the motifs found by MEME. Datasets are from (A) AG (B) AP1 (C) AP3 (D) PI (E) SEP3 (F) SOC1. (PDF 122 kb)

Additional file 11: **Figure S5.** G-box like motifs. Logo representation of all matches to the motifs found by MEME. Datasets are from (A) AG (B) AP3 (C) PI (D) SEP3 (E) SVP. (PDF 81 kb)

Additional file 12: **Figure S6.** TCP-like motifs. Logo representation of all matches to the motifs found by MEME. Datasets are from (A) AP1 (motif similar to TCP type II motif); (B) SEP3 (motif similar to TCP type I motif); (C) SEP3 (motif similar to TCP type II motif) and (D) SOC1 (motif similar to TCP type II motif). (PDF 65 kb)

Additional file 13: **Figure S7.** Central enrichment of G-boxes and TCP class I and II. Kernel density plot showing positions in peak centers relative to the peak summit of matches to the G-box (CACGTG) and the motifs for TCP class I (GGNCCCAC) and class II (GGGNCC(A/G)C) in (A) AG; (B) AP1; (C) AP3; (D) FLC; (E) PI; (F) SEP3; (G) SOC1 and (H) SVP. (PDF 232 kb)

Additional file 14: **Figure S8.** Heatmap of occurences of extensions of individual perfect CArG boxes. For each MADS TF, color indicates the percentage occurence of specific subsequences (per line). Red, zero occurence; the more yello*w*/white, the higher the percentage. (PDF 70 kb)

Additional file 15: **Table S7**. Top 5 most occurring extensions on the 5′ side and the 3′ side of the CArG-box and the top 5 combinations of 5′ and 3′ extensions. CArG-boxes were defined de novo by MEME-ChIP as described in the Material and Methods section. For each CArG-box like sequence, positions 1 and 10 were defined by comparing the sequence to the canonical CArG-box (CC(A/T)_6_GG. Position 1 was defined as the position that corresponds to the first C in the canonical CArG-box and position 10 was defined as the position that corresponds to the last G in the canonical CArG-box. Also, the strand defined by MEME-ChIP was taken to distinguish the 5′ and the 3′ sides. Subsequently, the three nucleotides on the 5′ side of position 1 and on the 3′ side of position 10 were counted for each CArG-box. The top 5 most occurring extensions and extension combinations were defined for (A) AG, (B) AP1, (C) AP3, (D) FLC, (E) PI, (F) SEP3, (G) SOC1 and (H) SVP. (PDF 95 kb)

Additional file 16: **Table S8.** Number of CArG-boxes with and without mutations. Note that the CArG-boxes in this table all match to the motif of AG because these CArG-boxes were used for all mutation analyses. The total number of CArG-boxes therefore differs from that in Additional file 5: Table S4. (PDF 47 kb)

Additional file 17: **Figure S9.** Conservation of CArG-boxes in ChIP-seq peaks among *Arabidopsis thaliana* ecotypes. (A) For each position in each occurrence of a CArG-box, color indicates entropy as a measure for conservation of that position among the ecotypes. Legend indicates logarithmic scale used for entropy values, between 0.0 (perfect conservation) and the maximum observed value of 0.4. CArG-box occurrences are ordered such that those with similar entropy values are close together. The white block observed at the bottom ~ one third of the plot indicates completely conserved CArG-box occurrences. (B) Relationship between entropy of a motif position, and mutation index in ecotypes. Each dot represent one positions of a CArG-box including a 3 nucleotide extension. Entropy for a motif position was obtained using all CArG-boxes underlying the motif logo in Col-0. Position 10 is plotted separately, as it is a major outlier; this is explained by the much stronger conservation obtained for this position in perfect CArG boxes. (PDF 55 kb)

## Abbreviations

AG: AGAMOUS
AP1: APETALA1
AP3: APETALA3
bHLH: basic helix-loop-helix (TF family)
bp: basepairs
bZIP: basic leucine zipper (TF family)
CArG: C-A/T-rich-G
ChIP-seq: Chromatin immunoprecipitation-sequencing
FDR: False Discovery Rate
FLC: FLOWERING LOCUS C
MADS: MCM1, AGAMOUS, DEFICIENS and SRF (TF family)
PI: PISTILLATA
PWM: Position Weight Matrix
SEP3: SEPALLATA3
SNP: Single Nucleotide Polymorphism
SOC1: SUPPRESSOR OF OVEREXPRESSION OF CONSTANS1
SVP: SHORT VEGETATIVE PHASE
TCP: TB1, CYC and PCF (TF family)
TF: Transcription factor
WRKY: TF family named after amino acid motif in core (WRKYGQK)
