# Supplementary material for: Comparative analysis of binding patterns of MADS-domain proteins in Arabidopsis thaliana
Source: BMC Plant Biol. 2018 Jun 25;18:131. doi: 10.1186/s12870-018-1348-8 (PMC6019531; doi:10.1186/s12870-018-1348-8)
Supplement: Supplementary file 1 — Table S1. Percentage of peak centers of which at least half the peak center falls within the promoter. (PDF 45 kb) [file 12870_2018_1348_MOESM1_ESM.pdf]

Dataset	Peak centers overlapping at least 50% with 500 bp upstream of TSS (%)	Peak centers overlapping at least 50% with 1000 bp upstream of TSS (%)	Peak centers overlapping at least 50% with 3000 bp upstream of TSS (%)
AG	41	53	78
AP1	27	40	70
AP3	40	51	75
FLC	49	59	80
PI	40	51	77
SEP3	41	53	77
SOC1	43	54	78
SVP	47	59	78
