# Supplementary material for: Comparative analysis of binding patterns of MADS-domain proteins in Arabidopsis thaliana
Source: BMC Plant Biol. 2018 Jun 25;18:131. doi: 10.1186/s12870-018-1348-8 (PMC6019531; doi:10.1186/s12870-018-1348-8)
Supplement: Supplementary file 2 — Table S2. Number of peaks in original analysis and after re-analysis. (PDF 45 kb) [file 12870_2018_1348_MOESM2_ESM.pdf]

**Dataset**	**Number of peaks in original analysis**	**Number of peaks after re-analysis**
AGAMOUS (AG)	1421	897
APETALA1 (AP1)	956	789
APETALA3 (AP3)	1524	1237
FLOWERING LOCUS C (FLC)	315	59
PISTILLATA (PI)	1852	2156
SEPALLATA3 (SEP3)	6843	4447
SUPPRESSOR OF OVEREXPRESSION OF CONSTANS1 (SOC1)	363	301
SHORT VEGETATIVE PHASE (SVP)	521	445
