# Supplementary figures and images for: Comparative analysis of binding patterns of MADS-domain proteins in Arabidopsis thaliana
Source: BMC Plant Biol. 2018 Jun 25;18:131. doi: 10.1186/s12870-018-1348-8 (PMC6019531; doi:10.1186/s12870-018-1348-8)

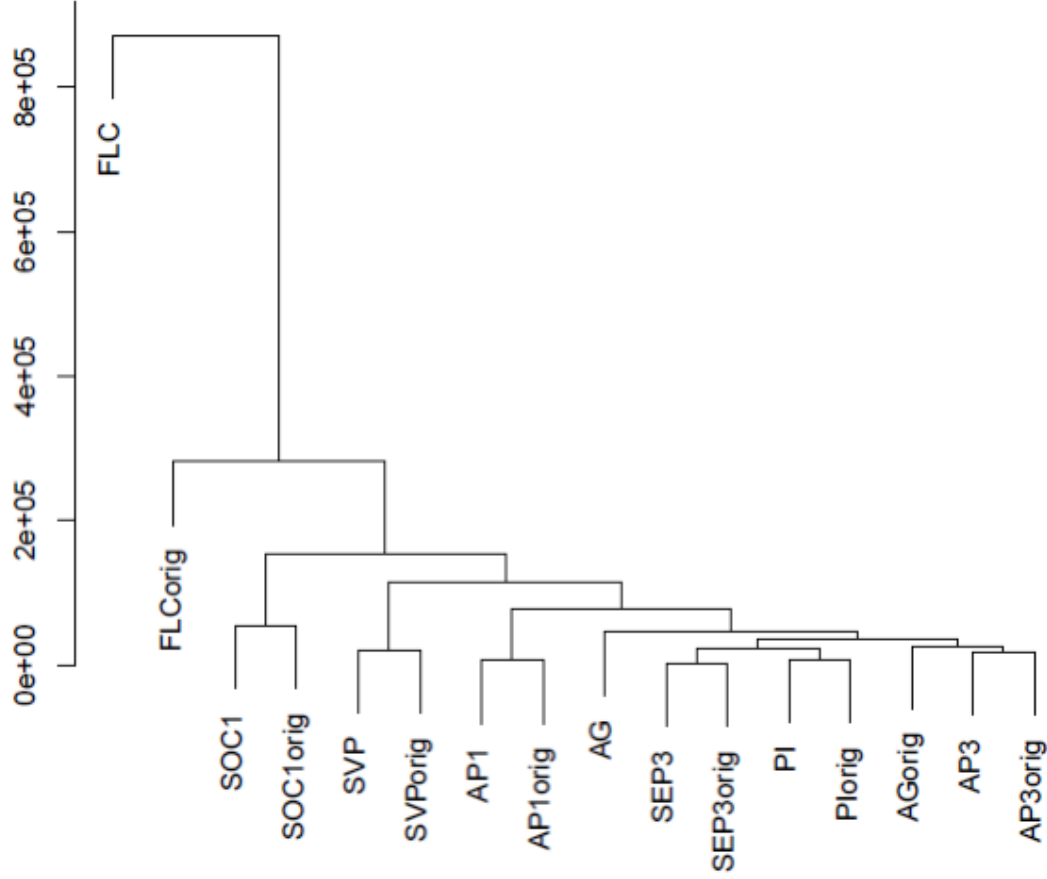

Supplement: Supplementary file 3 — Figure S1. Clustering of original peak sets and peaks sets after re-analysis. Hierarchical clustering with complete linkage (function hclust in R) was performed using as distance measure between two peak sets the average distance between peaks in one set and in the other. Peaks-sets after re-analysis are indicated with name of the transcription factor, original peak sets are indicated with the suffix “orig”. (PDF 38 kb) [file 12870_2018_1348_MOESM3_ESM.pdf]

**A**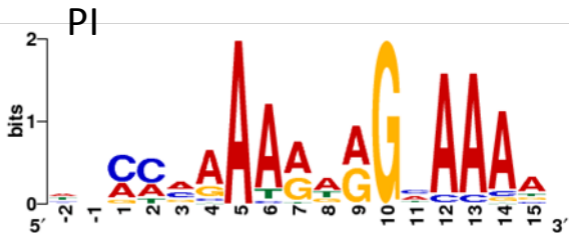**B**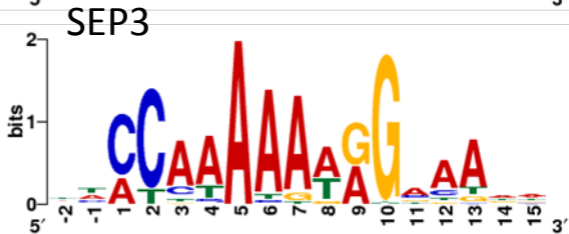**C**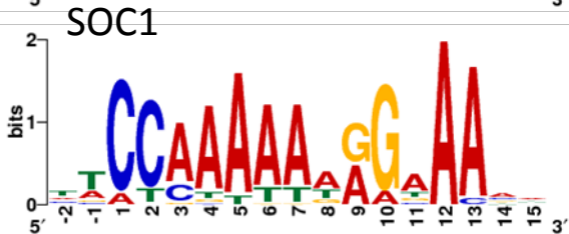

Supplement: Supplementary file 7 — Figure S2. CArG-box like motifs in unique peaks in PI, SEP3 and SOC1 datasets. Logo representation of all matches to the motif found by MEME. Logos are from (A) PI (B) SEP3 (C) SOC1. (PDF 126 kb) [file 12870_2018_1348_MOESM7_ESM.pdf]

# AG

**A**

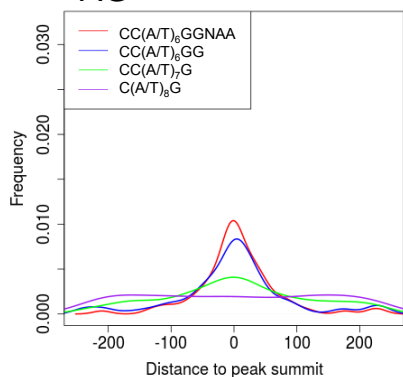

# AP1

**B**

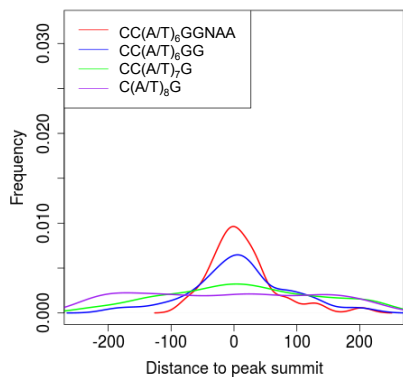

# AP3

**C**

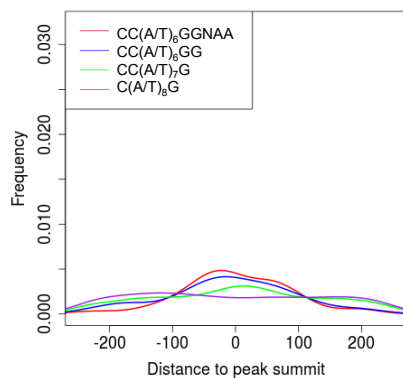

# FLC

**D**

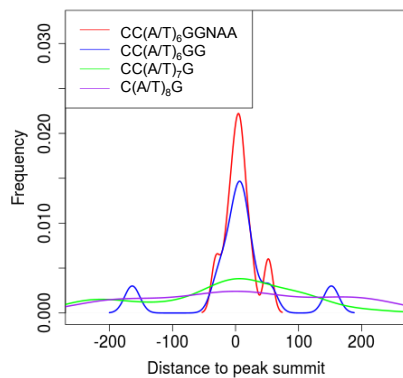

# PI

**E**

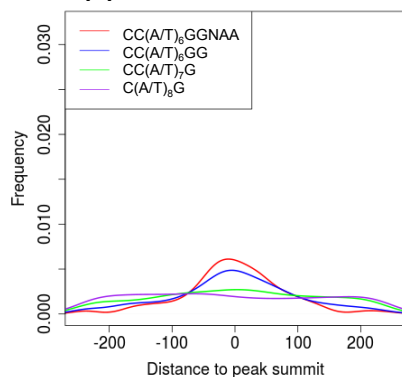

# SEP3

**F**

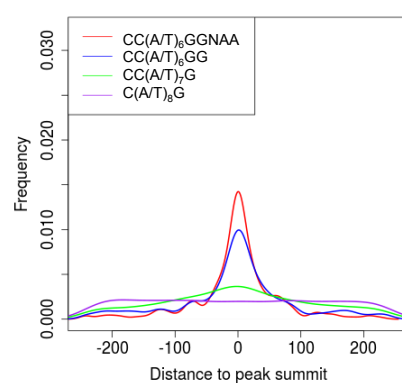

# SOC1

**G**

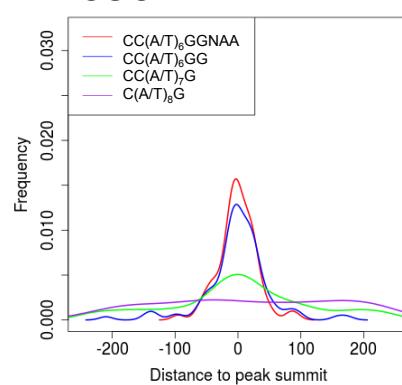

# SVP

**H**

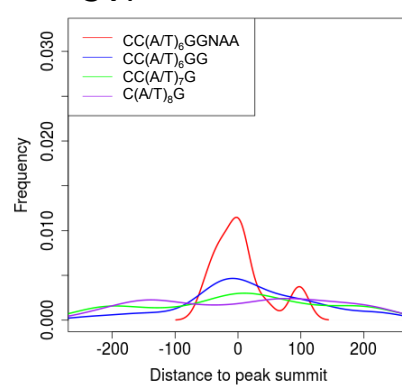

Supplement: Supplementary file 8 — Figure S3. Central enrichment of different CArG-box variants in different protein datasets. Kernel density plot of matches to the motif in peak centers relative to the peak summit. Plots are from (A) AG (B) AP1 (C) AP3 (D) FLC (E) PI (F) SEP3 (G) SOC1 (H) SVP. (PDF 251 kb) [file 12870_2018_1348_MOESM8_ESM.pdf]

AG

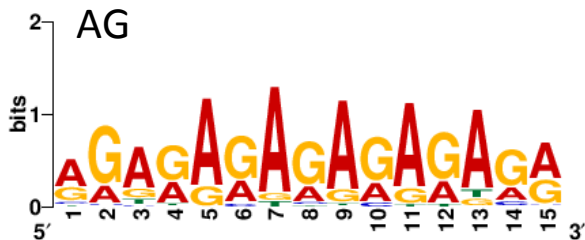

# AP1

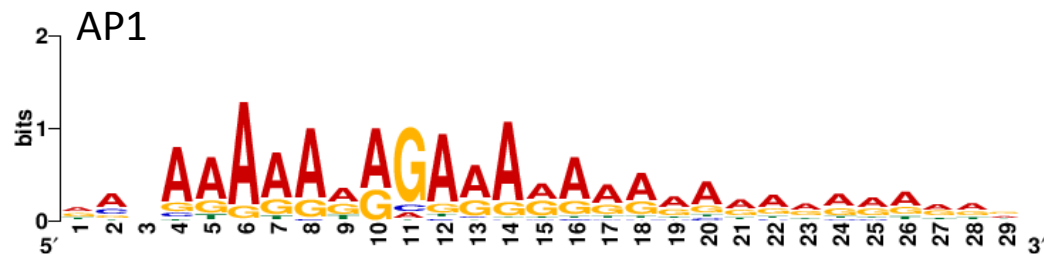

## AP3

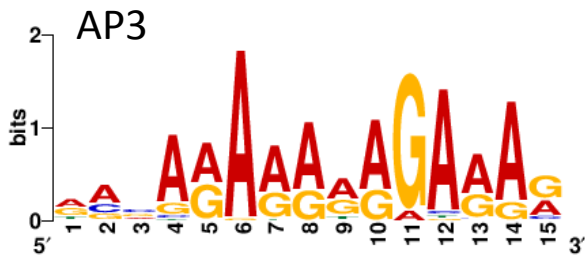

PI

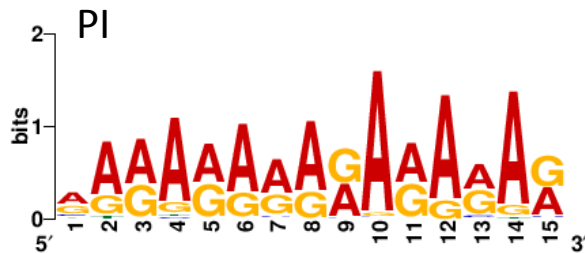

SEP3

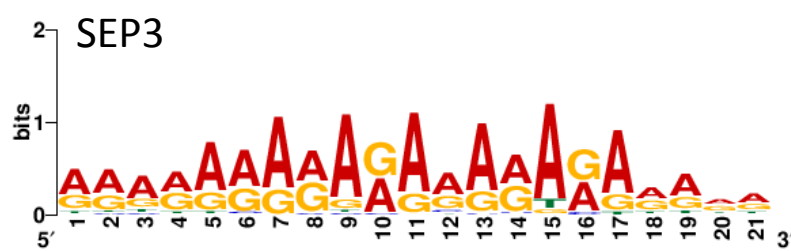

# SOC1

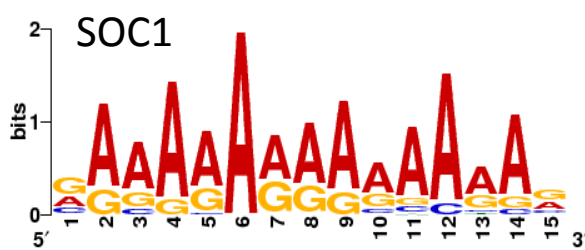

Supplement: Supplementary file 10 — Figure S4. GA/CT-rich motif. Logo representation of all matches to the motifs found by MEME. Datasets are from (A) AG (B) AP1 (C) AP3 (D) PI (E) SEP3 (F) SOC1. (PDF 122 kb) [file 12870_2018_1348_MOESM10_ESM.pdf]

**A**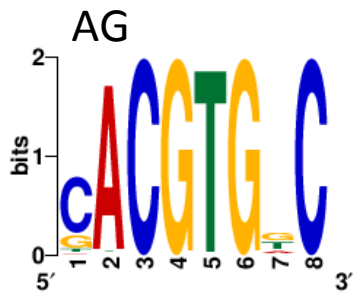**B**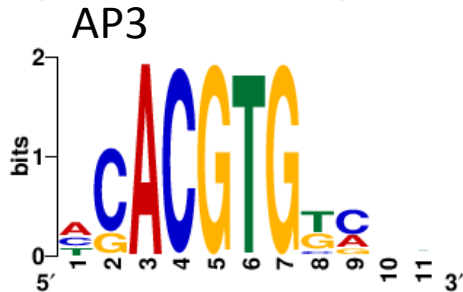**C**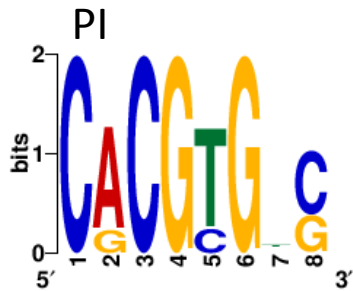**D**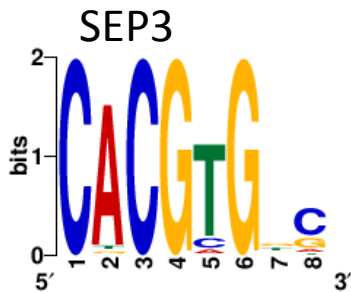**E**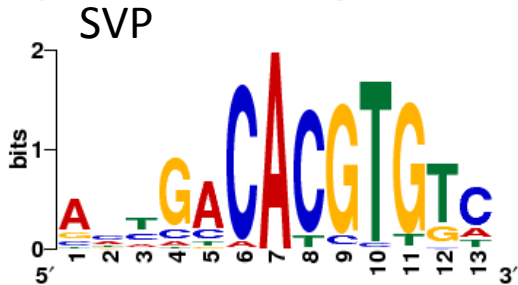

Supplement: Supplementary file 11 — Figure S5. G-box like motifs. Logo representation of all matches to the motifs found by MEME. Datasets are from (A) AG (B) AP3 (C) PI (D) SEP3 (E) SVP. (PDF 81 kb) [file 12870_2018_1348_MOESM11_ESM.pdf]

**A**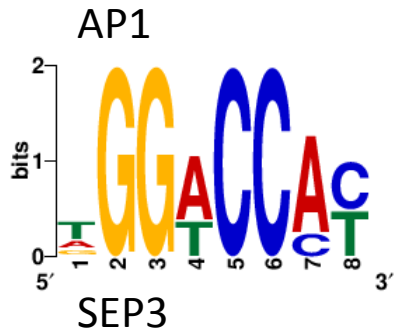**B**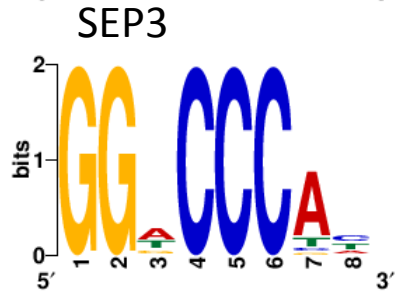**C**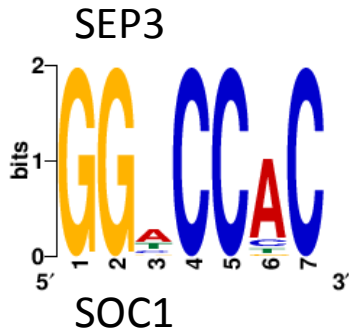**D**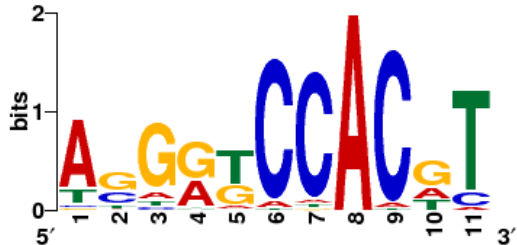

Supplement: Supplementary file 12 — Figure S6. TCP-like motifs. Logo representation of all matches to the motifs found by MEME. Datasets are from (A) AP1 (motif similar to TCP type II motif); (B) SEP3 (motif similar to TCP type I motif); (C) SEP3 (motif similar to TCP type II motif) and (D) SOC1 (motif similar to TCP type II motif). (PDF 65 kb) [file 12870_2018_1348_MOESM12_ESM.pdf]

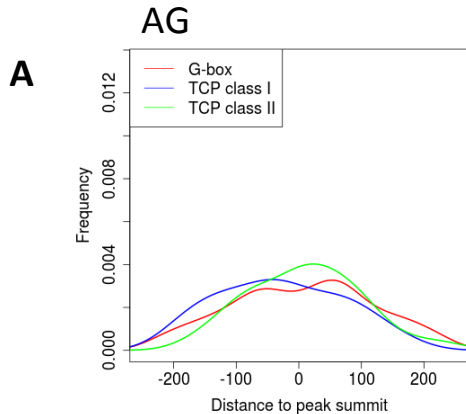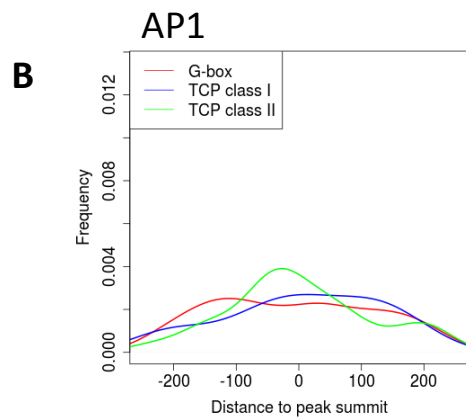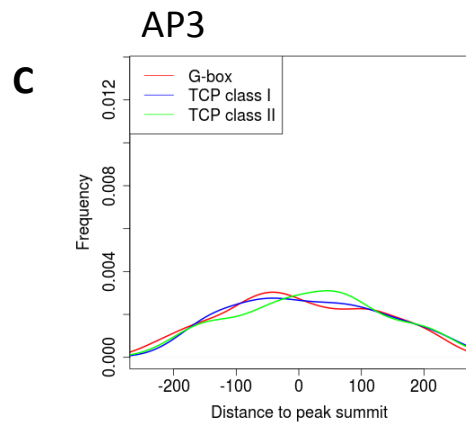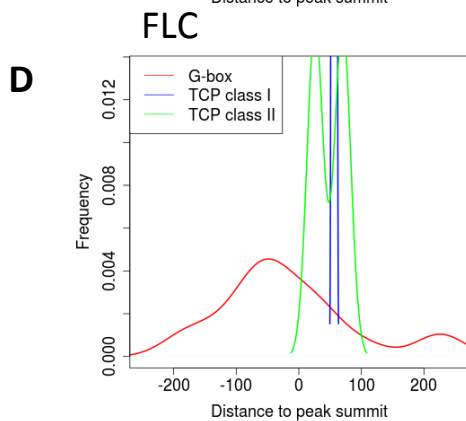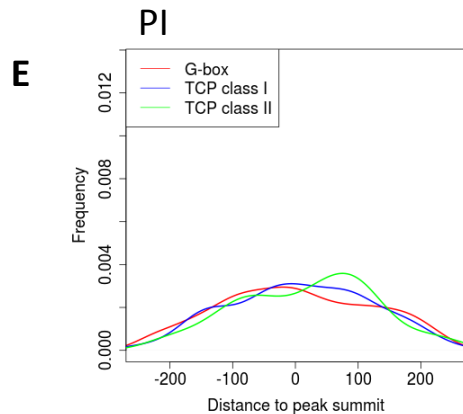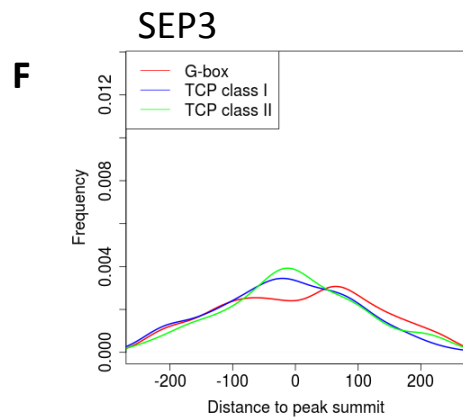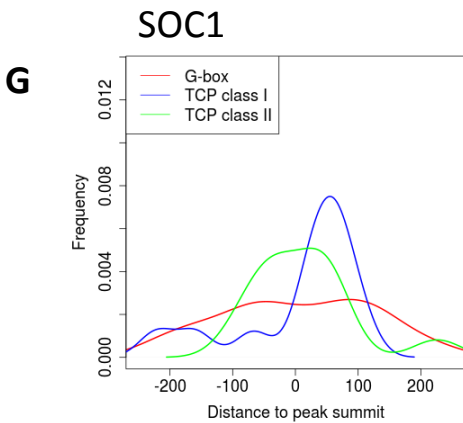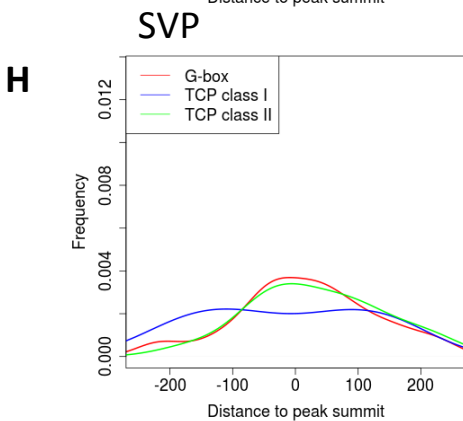

Supplement: Supplementary file 13 — Figure S7. Central enrichment of G-boxes and TCP class I and II. Kernel density plot showing positions in peak centers relative to the peak summit of matches to the G-box (CACGTG) and the motifs for TCP class I (GGNCCCAC) and class II (GGGNCC(A/G)C) in (A) AG; (B) AP1; (C) AP3; (D) FLC; (E) PI; (F) SEP3; (G) SOC1 and (H) SVP. (PDF 232 kb) [file 12870_2018_1348_MOESM13_ESM.pdf]

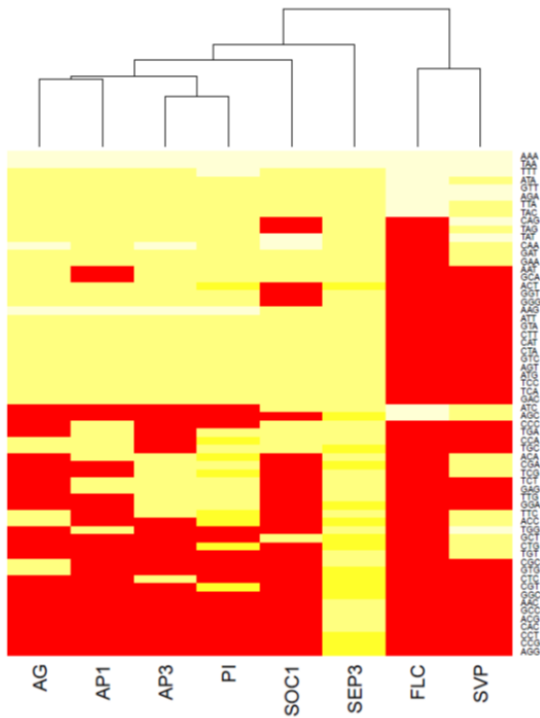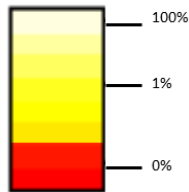

Supplement: Supplementary file 14 — Figure S8. Heatmap of occurences of extensions of individual perfect CArG boxes. For each MADS TF, color indicates the percentage occurence of specific subsequences (per line). Red, zero occurence; the more yellow/white, the higher the percentage. (PDF 70 kb) [file 12870_2018_1348_MOESM14_ESM.pdf]

**A**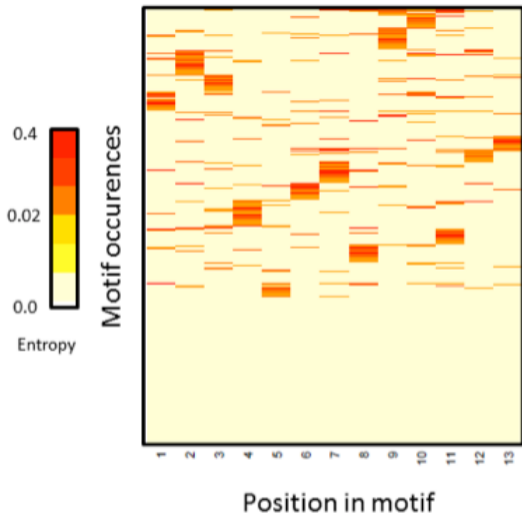**B**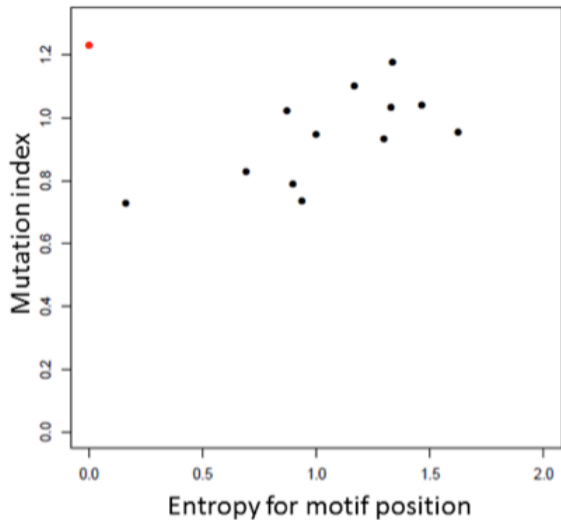

Supplement: Supplementary file 17 — Figure S9. Conservation of CArG-boxes in ChIP-seq peaks among Arabidopsis thaliana ecotypes. (A) For each position in each occurrence of a CArG-box, color indicates entropy as a measure for conservation of that position among the ecotypes. Legend indicates logarithmic scale used for entropy values, between 0.0 (perfect conservation) and the maximum observed value of 0.4. CArG-box occurrences are ordered such that those with similar entropy values are close together. The white block observed at the bottom ~ one third of the plot indicates completely conserved CArG-box occurrences. (B) Relationship between entropy of a motif position, and mutation index in ecotypes. Each dot represent one positions of a CArG-box including a 3 nucleotide extension. Entropy for a motif position was obtained using all CArG-boxes underlying the motif logo in Col-0. Position 10 is plotted separately, as it is a major outlier; this is explained by the much stronger conservation obtained for this position in perfect CArG boxes. (PDF 55 kb) [file 12870_2018_1348_MOESM17_ESM.pdf]
