# Supplementary material for: Comparative analysis of binding patterns of MADS-domain proteins in Arabidopsis thaliana
Source: BMC Plant Biol. 2018 Jun 25;18:131. doi: 10.1186/s12870-018-1348-8 (PMC6019531; doi:10.1186/s12870-018-1348-8)
Supplement: Supplementary file 4 — Table S3. Percentage of peaks in which motifs occur. For the CArG-box motifs, in addition to the percentage observed in all peaks, the percentage observed in the top 500 peaks is listed as well (between brackets). (PDF 55 kb) [file 12870_2018_1348_MOESM4_ESM.pdf]

Type of motif/ Figure / panel	Dataset	Percentage
**CArG-box (Figure 1)**		
A	AG	64% (69%)
B	AP1	30% (43%)
C	AP3	35% (57%)
D	FLC	76% (76%)
E	PI	42% (56%)
F	SEP3	47% (65%)
G	SOC1	83% (83%)
H	SVP	54% (54%)
**CArG-box like motifs in unique peaks (Additional file 7: Figure S2)**		
A	PI	35%
B	SEP3	38%
C	SOC1	78%
**GA/CT-rich motif (Additional file 10: Figure S4)**		
A	AG	50%
B	AP1	68%
C	AP3	29%
D	PI	64%
E	SEP3	61%
F	SOC1	67%
**G-box like motifs (Additional file 11: Figure S5)**		
A	AG	20%
B	AP3	27%
C	PI	25%
D	SEP3	21%
E	SVP	15%
**TCP-like motifs (Additional file 12: Figure S6)**		
A	AP1	30%
B	SEP3	15%
C	SEP3	17%
D	SOC1	16%
