# Supplementary material for: Comparative analysis of binding patterns of MADS-domain proteins in Arabidopsis thaliana
Source: BMC Plant Biol. 2018 Jun 25;18:131. doi: 10.1186/s12870-018-1348-8 (PMC6019531; doi:10.1186/s12870-018-1348-8)
Supplement: Supplementary file 5 — Table S4. Unique and overlapping CArG-boxes in each dataset. CArG-boxes are defined as all non-overlapping CArG-box like motifs found for each of the eight proteins (see Fig. 1 for corresponding motifs). Note that the amount of CArG-boxes occurring in all datasets is not the same for each dataset. This is caused by the fact that that the CArG-box definition obtained by the de novo motif search differs between the datasets. (PDF 50 kb) [file 12870_2018_1348_MOESM5_ESM.pdf]

Protein	Unique CArG-boxes	CArG-boxes also occurring in:							Total	Percentage unique CArG-boxes
		one other dataset	two other datasets	three other datasets	four other datasets	five other datasets	six other datasets	all other datasets[a]		
AG	34	95	154	233	190	55	15	11	787	4.3
AP1	15	56	57	54	71	32	6	5	296	5.1
AP3	30	76	148	141	121	26	8	5	555	5.4
FLC	2	11	13	9	10	4	7	12	68	2.9
PI	164	262	276	221	169	43	17	10	1162	14.1
SEP3	1311	525	374	281	204	50	18	10	2773	47.2
SOC1	54	83	67	43	70	47	17	11	392	13.8
SVP	72	33	13	17	9	9	13	10	176	40.9
