# Supplementary material for: Comparative analysis of binding patterns of MADS-domain proteins in Arabidopsis thaliana
Source: BMC Plant Biol. 2018 Jun 25;18:131. doi: 10.1186/s12870-018-1348-8 (PMC6019531; doi:10.1186/s12870-018-1348-8)
Supplement: Supplementary file 6 — Table S5. Unique and overlapping peak centers in each dataset. A peak center is defined as the region 250 bp upstream and downstream of a peak summit. (PDF 52 kb) [file 12870_2018_1348_MOESM6_ESM.pdf]

Protein	Unique peak centers	Peak centers overlapping with:							Total	% unique peaks
		one other dataset	two other datasets	three other datasets	four other datasets	five other datasets	six other datasets	all datasets		
AG	28	111	168	285	242	45	13	5	789	3.1
AP1	44	213	122	156	199	40	10	5	897	5.6
AP3	85	192	327	341	236	41	10	5	1237	6.9
FLC	5	11	9	8	9	6	6	5	59	8.5
PI	372	597	494	387	245	44	12	5	2156	17.3
SEP3	2318	870	554	396å	247	44	13	5	4447	52.1
SOC1	38	39	54	35	47	41	12	5	301	12.6
SVP	282	67	27	22	22	9	11	5	445	63.4
