# Supplementary material for: Comparative analysis of binding patterns of MADS-domain proteins in Arabidopsis thaliana
Source: BMC Plant Biol. 2018 Jun 25;18:131. doi: 10.1186/s12870-018-1348-8 (PMC6019531; doi:10.1186/s12870-018-1348-8)
Supplement: Supplementary file 9 — Table S6. Co-occurrence of several CArG-box variants in ChIP-seq peaks. (A) Amount of peaks with a single occurrence and with multiple occurrences of pre-defined CArG-box variants. (B) Amount of peaks that contain only CC(A/T)6GG, only CC(A/T)7G or both motifs. (C) Amount of peaks that contain only CC(A/T)7G, only C(A/T)8G or both motifs. (D) Amount of peaks that contain only CC(A/T)6GG, only C(A/T)8G or both motifs. Expected values for B, C and D were calculated by multiplying the frequencies of peaks with each motif and multiplying that with the total amount of peaks of a dataset. (PDF 71 kb) [file 12870_2018_1348_MOESM9_ESM.pdf]

A

Dataset	CC(A/T)$_6$GG		CC(A/T)$_7$GG		C(A/T)$_8$G	
	Single occurrence	Multiple occurrences	Single occurrence	Multiple occurrences	Single occurrence	Multiple occurrences
AG	139	9	256	53	262	100
AP1	90	8	221	48	244	93
AP3	123	3	296	58	339	100
FLC	9	1	13	8	15	9
PI	202	12	463	97	596	169
SEP3	475	21	1120	243	1354	443
SOC1	85	10	87	30	87	30
SVP	27	3	86	27	126	53

B

Dataset	CC(A/T)$_6$GG	CC(A/T)$_7$G	Together	Expected
AG	107	268	41	51
AP1	66	237	32	33
AP3	89	317	37	36
FLC	8	19	2	4
PI	162	508	52	56
SEP3	347	1214	149	152
SOC1	72	94	23	37
SVP	26	109	4	8

C

Dataset	CC(A/T)$_7$G	C(A/T)$_8$G	Together	Expected
AG	191	244	118	125
AP1	172	240	97	115
AP3	209	294	145	126
FLC	13	16	8	9
PI	353	558	207	199
SEP3	827	1261	536	551
SOC1	74	74	43	45
SVP	59	125	54	45

D

Dataset	CC(A/T)$_6$GG	C(A/T)$_8$G	Together	Expected
AG	90	304	58	60
AP1	53	292	45	42
AP3	75	388	51	45
FLC	8	22	2	4
PI	136	687	78	76
SEP3	298	1599	198	200
SOC1	60	82	35	37
SVP	21	170	9	12
