# Supplementary material for: Comparative analysis of binding patterns of MADS-domain proteins in Arabidopsis thaliana
Source: BMC Plant Biol. 2018 Jun 25;18:131. doi: 10.1186/s12870-018-1348-8 (PMC6019531; doi:10.1186/s12870-018-1348-8)
Supplement: Supplementary file 15 — Table S7. Top 5 most occurring extensions on the 5′ side and the 3′ side of the CArG-box and the top 5 combinations of 5′ and 3′ extensions. CArG-boxes were defined de novo by MEME-ChIP as described in the Material and Methods section. For each CArG-box like sequence, positions 1 and 10 were defined by comparing the sequence to the canonical CArG-box (CC(A/T)6GG. Position 1 was defined as the position that corresponds to the first C in the canonical CArG-box and position 10 was defined as the position that corresponds to the last G in the canonical CArG-box. Also, the strand defined by MEME-ChIP was taken to distinguish the 5′ and the 3′ sides. Subsequently, the three nucleotides on the 5′ side of position 1 and on the 3′ side of position 10 were counted for each CArG-box. The top 5 most occurring extensions and extension combinations were defined for (A) AG, (B) AP1, (C) AP3, (D) FLC, (E) PI, (F) SEP3, (G) SOC1 and (H) SVP. (PDF 95 kb) [file 12870_2018_1348_MOESM15_ESM.pdf]

A) AG

**Rank**	5’ ex-tension	Occur-rence	3’ ex-tension	Occur-rence	5’ + 3’ extension combination	Occur-rence	Ex-pected occur-rence	Observed / expected
1	TTT	71	AAA	357	5'-TTT + 3'-AAA	31	32.207	0.963
2	TTA	59	CAA	95	5'-TTA + 3'-AAA	27	26.764	1.009
3	AAA	46	TAA	94	5'-AAA + 3'-AAA	22	20.867	1.054
4	TTG	39	AAG	48	5'-TTG + 3'-AAA	21	17.691	1.187
5	ATA	35	GAA	28	5'-ATA + 3'-AAA	16	15.877	1.008

B) AP1

**Rank**	5’ ex-tension	Occur-rence	3’ ex-tension	Occur-rence	5’ + 3’ extension combination	Occur-rence	Ex-pected occur-rence	Observed / expected
1	TTT	29	AAA	49	5'-TTA + 3'-AAA	5	3.311	1.51
2	TTA	20	CAA	15	5'-TTT + 3'-TAA	4	1.372	2.916
3	ATA	12	TAA	14	5'-TTT + 3'-AAA	4	4.801	0.833
4	CTT	11	ATA	12	5'-AAT + 3'-AAA	4	1.159	3.452
5	AAA	11	AAG	11	5'-TAT + 3'-AAA	3	0.828	3.624

C) AP3

**Rank**	5’ ex-tension	Occur-rence	3’ ex-tension	Occur-rence	5’ + 3’ extension combination	Occur-rence	Ex-pected occur-rence	Observed / expected
1	AAA	43	AAA	186	5'-AAA + 3'-AAA	17	14.411	1.18
2	TTT	39	TAA	83	5'-TTT + 3'-AAA	16	13.07	1.224
3	TTA	28	CAA	65	5'-TTA + 3'-AAA	12	9.384	1.279

4	AAT	24	GAA	36	5'-AAT + 3'-AAA	11	8.043	1.368
5	TTG	19	ATA	31	5'-TTG + 3'-AAA	9	6.368	1.413

D) FLC

**Rank**	5’ extension	Occurrence	3’ extension	Occurrence	5’ + 3’ extension combination	Occurrence	Expected occurrence	Observed / expected
1	TTT	10	AAA	52	5'-TTT + 3'-AAA	6	7.647	0.785
2	ATT	5	TAA	12	5'-ATT + 3'-AAA	4	3.824	1.046
3	TTG	3	AAT	3	5'-TTT + 3'-TAA	3	1.765	1.7
4	TTA	3	CAA	1	5'-TTG + 3'-AAA	3	2.294	1.308
5	ATA	3	-	-	5'-TTA + 3'-AAA	3	2.294	1.308

E) PI

**Rank**	5’ extension	Occurrence	3’ extension	Occurrence	5’ + 3’ extension combination	Occurrence	Expected occurrence	Observed / expected
1	TTT	135	AAA	465	5'-TTT + 3'-AAA	49	54.023	0.907
2	TTA	109	TAA	133	5'-TTA + 3'-AAA	42	43.619	0.963
3	ATT	57	CAA	109	5'-AAA + 3'-AAA	25	22.41	1.116
4	AAA	56	AAG	70	5'-ATA + 3'-AAA	24	20.409	1.176
5	ATA	51	ATA	53	5'-AAT + 3'-AAA	21	16.407	1.28

F) SEP3

**Rank**	5’ extension	Occurrence	3’ extension	Occurrence	5’ + 3’ extension combination	Occurrence	Expected occurrence	Observed / expected
1	TTT	193	AAA	1301	5'-TTT + 3'-	92	90.549	1.016

					AAA			
2	AAA	179	CAA	257	5'-AAA + 3'-AAA	90	83.981	1.072
3	TTA	157	TAA	239	5'-TTA + 3'-AAA	74	73.659	1.005
4	ATA	104	AAG	164	5'-ATA + 3'-AAA	52	48.793	1.066
5	ATT	84	GAA	127	5'-AAT + 3'-AAA	45	39.41	1.142

G) SOC1

**Rank**	5’ extension	Occurrence	3’ extension	Occurrence	5’ + 3’ extension combination	Occurrence	Expected occurrence	Observed / expected
1	TTT	78	AAA	229	5'-TTT + 3'-AAA	36	45.566	0.79
2	TTA	42	TAA	40	5'-TTA + 3'-AAA	22	24.536	0.897
3	ATT	27	CAA	33	5'-TTG + 3'-AAA	15	12.268	1.223
4	ATA	22	GAA	15	5'-ATT + 3'-AAA	12	15.773	0.761
5	TTG	21	AAT	14	5'-AAT + 3'-AAA	11	9.347	1.177

H) SVP

**Rank**	5’ extension	Occurrence	3’ extension	Occurrence	5’ + 3’ extension combination	Occurrence	Expected occurrence	Observed / expected
1	TTT	18	AAA	119	5'-TTT + 3'-AAA	12	12.17	0.986
2	AAT	9	TAA	32	5'-AAA + 3'-AAA	8	6.085	1.315
3	AAA	9	AAG	6	5'-AAT + 3'-AAA	7	6.085	1.15
4	TTG	7	AAT	4	5'-TTG + 3'-AAA	6	4.733	1.268
5	TGA	7	TCA	3	5'-TCT + 3'-AAA	5	4.733	1.056
