# Supplementary material for: Comparative analysis of binding patterns of MADS-domain proteins in Arabidopsis thaliana
Source: BMC Plant Biol. 2018 Jun 25;18:131. doi: 10.1186/s12870-018-1348-8 (PMC6019531; doi:10.1186/s12870-018-1348-8)
Supplement: Supplementary file 16 — Table S8. Number of CArG-boxes with and without mutations. Note that the CArG-boxes in this table all match to the motif of AG because these CArG-boxes were used for all mutation analyses. The total number of CArG-boxes therefore differs from that in Additional file 5: Table S4. (PDF 47 kb) [file 12870_2018_1348_MOESM16_ESM.pdf]

Dataset	Number of CArG-boxes without mutations	Number of CArG-boxes with mutations	Total
AG	294	493	787
AP1	208	281	489
AP3	282	501	783
FLC	26	28	54
PI	494	836	1330
SEP3	909	1640	2549
SOC1	126	236	362
SVP	69	99	168
Combined	1459	2883	4342
